# Molecular Principles of Proton-Coupled Quinone Reduction in the Membrane-Bound Superoxide Oxidase

**DOI:** 10.1021/jacs.4c17055

**Published:** 2025-02-12

**Authors:** Daniel Riepl, Abbas Abou-Hamdan, Jonas Gellner, Olivier Biner, Dan Sjöstrand, Martin Högbom, Christoph von Ballmoos, Ville R. I. Kaila

**Affiliations:** † Department of Biochemistry and Biophysics, The Arrhenius Laboratories for Natural Sciences, Stockholm University, SE-106 91 Stockholm, Sweden; ‡ Department of Chemistry, Biochemistry and Pharmaceutical Sciences, University of Bern, CH-3012 Bern, Switzerland; § Department of Chemistry, Technical University Munich, D-85748 Garching, Germany

## Abstract

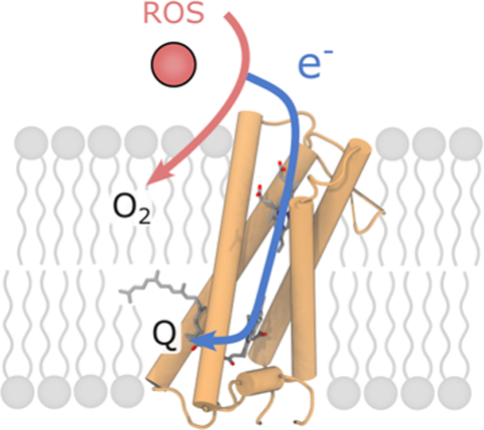

Reactive oxygen species (ROS) are physiologically harmful radical species generated as byproducts of aerobic respiration. To detoxify ROS, most cells employ superoxide scavenging enzymes that disproportionate superoxide (O_2_^·–^) to oxygen (O_2_) and hydrogen peroxide (H_2_O_2_). In contrast, the membrane-bound superoxide oxidase (SOO) is a minimal 4-helical bundle protein that catalyzes the direct oxidation of O_2_^·–^ to O_2_ and drives quinone reduction by mechanistic principles that remain unknown. Here, we combine multiscale hybrid quantum/classical (QM/MM) free energy calculations and microsecond molecular dynamics simulations with functional assays and site-directed mutagenesis experiments to probe the mechanistic principles underlying the charge transfer reactions of the superoxide-driven quinone reduction. We characterize a cluster of charged residues at the periplasmic side of the membrane that functions as a O_2_^·–^ collecting antenna, initiating electron transfer via two *b* hemes to the active site for quinone reduction at the cytoplasmic side. Based on multidimensional QM/MM string simulations, we find that a proton-coupled electron transfer (PCET) reaction from the active site heme *b* and nearby histidine residues (H87, H158) results in quinol (QH_2_) formation, followed by proton uptake from the cytoplasmic side of the membrane. The functional relevance of the identified residues is supported by site-directed mutagenesis and activity assays, with mutations leading to inhibition of the O_2_^·–^-driven quinone reduction activity. We suggest that the charge transfer reactions could build up a proton motive force that supports the bacterial energy transduction machinery, while the PCET machinery provides unique design principles of a minimal oxidoreductase.

## Introduction

Aerobic life relies on the oxidation of nutrients and reduction of oxygen to water to generate an electrochemical proton motive force (PMF) across a biological membrane that drives the synthesis of adenosine triphosphate (ATP).^1^ The underlying charge transfer reactions are catalyzed by the respiratory chain complexes that stepwise shuttle the electrons from nicotine amide adenine dinucleotide (NADH, *E*^0^ = −320 mV vs NHE) to O_2_ (*E*^0^ = +820 mV) and couple this process to proton translocation across the membrane.^2^

Certain reversible reactions in the respiratory chains, particularly in complexes I and III, can result in a reverse electron transfer (RET) and formation of reactive oxygen species (ROS) by the reduction of oxygen (O_2_) to superoxide (O_2_^·–^, *E*^0^ = −160 mV).^3−6^ O_2_^·–^ can be further reduced to produce other physiologically harmful molecules, such as hydrogen peroxide (H_2_O_2_) or hydroxyl radicals (OH^·^). Owing to their high reactivity, elevated concentrations of ROS damage cells by oxidizing nucleic acids and lipids, and inhibiting various enzymes.^3^ An increased ROS production resulting, e.g., from mutations of the respiratory chain enzymes, has been implicated in various mitochondrial diseases,^7^ while ROS formation is also used by the immune system to kill pathogens^8,9^ as well as in cell signaling.^10,11^ A careful regulation of cellular ROS levels is thus essential, and must be tightly controlled by specialized enzymes that metabolize and remove unwanted ROS. These enzymes often exhibit rate constants in the diffusion-limited regime, which, together with a high protein concentration as compared to superoxide, results in significantly faster ROS scavenging relative to the spontaneous dismutation reaction (2O_2_^·–^ + 2H^+^ → O_2_ + H_2_O_2_) at physiological conditions.^12^

Superoxide is commonly removed by various isoforms of superoxide dismutase (SOD)^13−15^ found in most cellular compartments, catalyzing the disproportionation of O_2_^·–^ into O_2_ and H_2_O_2_. The latter is subsequently reduced by catalase or peroxidase,^16^ while superoxide reductase (SOR) reduces cytoplasmic superoxide into hydrogen peroxide^17^ (O_2_^·–^ + 2H^+^ + e^–^ → H_2_O_2_) in anaerobic bacteria. Interestingly, while these enzymes have evolved independently, they all employ similar metal-catalyzed redox reactions with Cu^1+/2+^, Zn^2+^, Ni^2+/3+^, Mn^2+/3+^, or nonheme Fe^2+/3+^ ions to catalyze the reactions.

**Figure 1:**
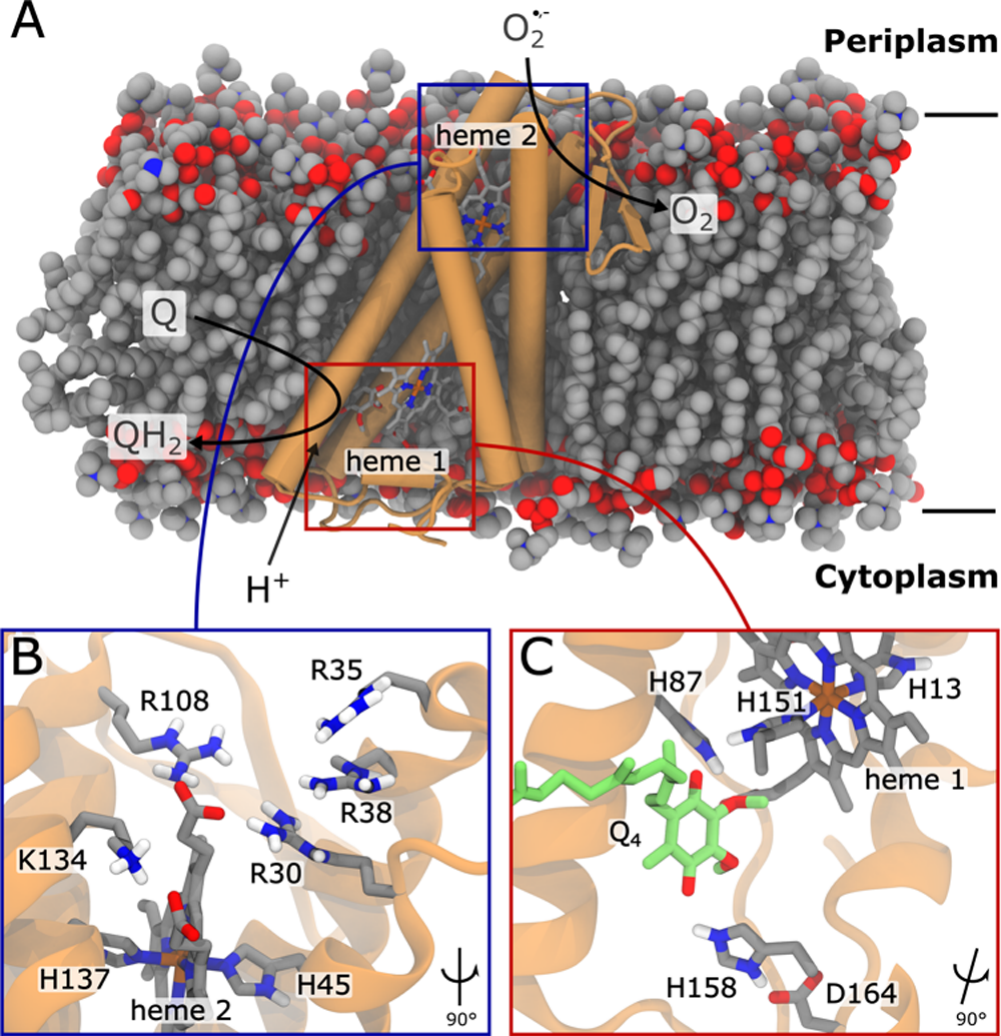
Structure and function of superoxide oxidase. (A) Overview of the structure of superoxide oxidase (SOO), modeled in a membrane. SOO couples superoxide oxidation to quinone reduction via two heme *b* cofactors (heme 1 and 2). (B) The putative superoxide binding site is located on the P-side of the membrane close to the heme 2 cofactor and comprises several positively charged residues. (C) The quinone binding site, near heme 1 on the N-side of the membrane, contains several histidine residues that form hydrogen bonds with the modeled quinone (Q_4_) species.

Recently, a new superoxide scavenging enzyme, superoxide oxidase (SOO) or CybB, was isolated from *E. coli*(18) and its structure was determined by X-ray crystallography (cf. also refs (19,20)). In contrast to the soluble SOD and SOR, SOO (Figure 1) is a membrane-bound enzyme that comprises only four transmembrane helices with two *b*-type hemes that transfer the electrons from superoxide to quinone (Q). The removal of electrons from periplasmic superoxide and protons from the cytoplasm to form molecular oxygen and reduced quinol, suggests that SOO could generate a PMF across the membrane and contribute to the energy transduction machinery by recovering some of the redox energy lost from the generation of ROS, although the bioenergetics of the process remain unclear. Previous studies^18,21^ revealed a reversible superoxide:quinone oxidoreductase activity with both oxidized and reduced ubiquinone and menaquinone as viable substrates, and putative binding sites for superoxide and Q, suggested based on structural data. Redox midpoint potentials for the heme *b* cofactors were determined at −23/+48 mV for the *apo* state and −8/+100 mV (vs NHE) in the presence of Q,^21^ and thus compatible with those of both superoxide (−160 mV) and quinone (+90 mV ubiquinone/–80 mV menaquinone) at standard conditions (see Discussion).

Despite these insights, the proton-coupled electron transfer (PCET) reactions underlying the superoxide-driven quinone reduction process remain unclear. Here we probe the molecular mechanisms underlying these charge transfer reactions by combining multiscale molecular simulations^22^ with site-directed mutagenesis experiments and functional studies. To probe the free energy landscape of the PCET reactions along the multidimensional reaction pathways, we develop a hybrid quantum/classical (QM/MM) string stimulation method that reveals important proton and electron donors employed in the quinone reduction process, while our functional assays validate our mechanistic predictions. Our combined findings provide functional insight into SOO as a unique minimal energy-transducing oxidoreductase.

## Results

### Principles of Superoxide Binding

**Figure 2:**
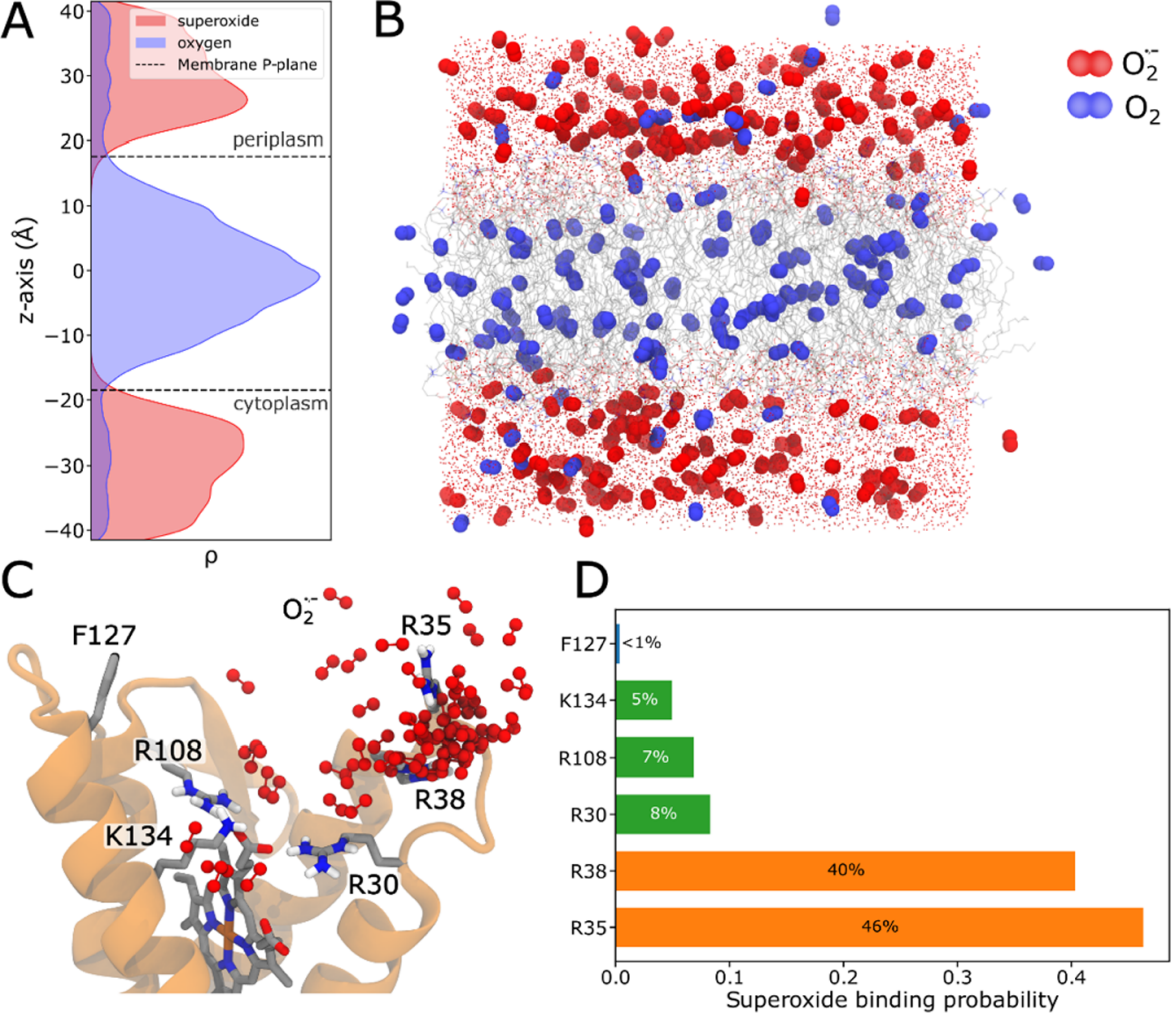
Principles of superoxide binding. (A) Oxygen and superoxide distribution, perpendicular to the membrane plane, during microsecond MD simulations (simulations S1, S2). The phosphorus atoms of the lipids are marked by dotted lines. Molecular oxygen partitions predominantly into the membrane, while the superoxide is found in the bulk water and at the membrane/water interface. The geometric center of the membrane is defined at *z* = 0 Å. (B) Ensemble-averaged image of the oxygen (in blue) and superoxide (in red) positions during the MD simulations (simulation S6, S2). (C) Overlay of superoxide binding positions using 100 evenly spaced MD frames (simulation S1) and (D) calculated superoxide binding probabilities for the charged residues in the pocket. R35 and R38 show the highest superoxide occupancy and act as antennas that collect superoxide from the bulk. From the R35/R38 site, the superoxide could further diffuse to the smaller cluster near R30/R108 and K134, which would enable fast electron transfer to heme 2. The latter residues also interact with the propionic groups of heme 2 and could shield the superoxide from the negative charge of the latter. F127 was used as a probe site in the same region to account for random diffusion relative to proposed superoxide binding residues.

To probe the mechanism of the superoxide binding, we performed over 30 μs of classical molecular dynamics (MD) simulations of SOO embedded in a lipid membrane (Figure S1), with either 10 superoxide or oxygen molecules initially solvated in the bulk phase. During the MD simulations, a large fraction of molecular oxygen partitions into the hydrophobic membrane core, with ca. 12 times more O_2_ molecules in the core of the membrane relative to the bulk, consistent with the 5–10 higher solubility of O_2_ in membranes as compared to water^23−29^ (Figure 2A,B). In contrast, the anionic superoxide localizes into the bulk water phase and at the water-membrane interface, close to the lipid headgroups or at a cationic binding site on the periplasmic side (P-side) of SOO (see below, Figure 2A). We obtain similar superoxide distributions in simulations with both a pure POPC membrane and with an *E. coli* polar lipid mixture (75% POPE, 20% POPG, 5% cardiolipin) (Figure S2A), suggesting that the overall results are robust. The overall structure of SOO remains similar in the MD simulations as compared to the detergent-solubilized (DDM) X-ray structure of SOO (RMSD < 2.5 Å, Figure S3), although we observe a subtle opening of the helical bundle and a transient interaction between the C-terminal horizontal helical stretch and the phospholipid headgroups in the MD simulations (Figure S2B). The dynamics of SOO extracted from the MD trajectories resemble the experimental B-factors (Figure S4A), suggesting that the simulations correctly capture the motions of the enzyme in the membrane. We observe a high degree of flexibility in the periplasmic loop, containing R35 and R38, as well as in the cytoplasmic C-terminal loop, while the core of the protein remains rather rigid. Interestingly, the mobility of the short cytoplasmic helical segment, orientated perpendicularly to the membrane, and the C-terminal loop is sensitive to the conformation of R169 (Figure S4B,C).

The superoxide accumulates in the MD simulations around a positively charged cavity on the periplasmic side of SOO, comprising R30, R35, R38, R108, and K134 (Figures 1B and S5, Movie 1). In this regard, we find a dominant superoxide occupancy around R35 and R38 (ca. 40% occupation over the complete simulation trajectories) that serve as superoxide-collecting antennas, and reach into the bulk solvent to attract superoxide molecules into electron transfer distance from heme 2 (Figure 2D). Moreover, another local docking site forms around R30, R108, and K134 (10% total occupation) (Figure 2C,D) near the propionic groups of heme 2.

**Figure 3:**
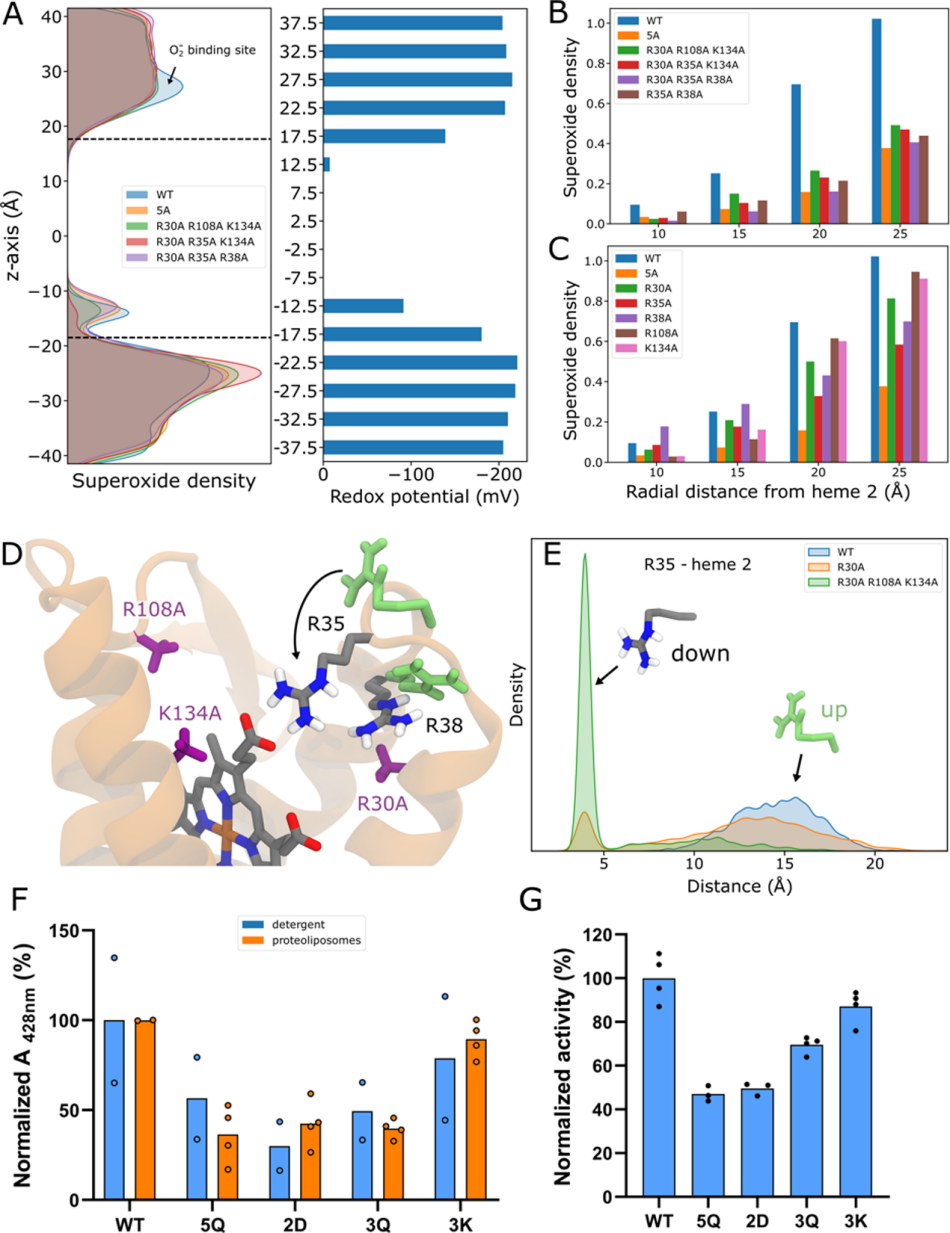
Engineering the superoxide binding site. (A) Left: overall superoxide distribution perpendicular to the membrane plane (*z*-axis). Comparing the WT to the quintuple and triple mutants, shows loss of superoxide binding at *z* = +25 Å near the putative binding site. The geometric center of the membrane is defined at *z* = 0 Å. Right: shift in oxygen/superoxide redox potential due to concentration changes along the membrane axis (−160 mV + 59 mV × log_10_([O_2_^·–^]/[O_2_])). The distributions are nonsymmetric due to the depletion of superoxide from the periplasmic side in the MD simulations (see also Methods). (B, C) Radial distribution of the local superoxide density relative to heme 2 (atom C2D) in the WT and in silico variants. Quintuple and triple mutants show significantly lower superoxide concentrations. In contrast, single-point mutants show similar behavior as the WT, with the exception of R30A and R38A that lead to a flip of R35 to compensate for the mutation. See also Supplementary Figure S5 for detailed radial distribution function profiles. (D) Variants with multiple substitutions in the proximal R30/R108/K134 site, show a “down” flip of R35 that forms a contact with the propionic group of heme 2, while R35 is in an “up” conformation in the WT. (E) Comparison of the heme 2 propionate – R35 distance shows a small shift in the R30A single mutant, but a drastic shift in the triple mutant relative to the WT. Selected snapshots of the two conformations are shown in panel D. (F) Equilibrium reduction level of SOO heme content in different superoxide binding site variants upon exposure to a superoxide producing xanthine oxidase/hypoxanthine system. SOO was either present as solubilized protein or reconstituted into proteoliposomes. 5Q – R30Q/R35Q/R38Q/R108Q/K134Q; 2D – R30D/R108D; 3Q – R30Q/R108Q/K134Q; 3K – R30K/R108 K/K134R. (G) Normalized steady state activity of the forward reaction by measuring the decrease in formazan rate formation upon addition of 100 nM of different variants of SOO (see Figure S7). 5Q – R30Q/R35Q/R38Q/R108Q/K134Q; 2D – R30D/R108D; 3Q – R30Q/R108Q/K134Q; 3K – R30K/R108 K/K134R.

To probe the importance of the identified residues, we next replaced R30, R35, R38, R108, and K134 by alanine or asparagine residues first in silico (see Table S1), followed by site-directed mutagenesis experiments (see below). In simulations of the wildtype (WT) SOO, we observe a high accumulation of superoxide near heme 2 (10%/3% of the total O_2_^·–^ within 25 Å/15 Å edge-to-edge distance, Figures 3B,C and S6), consistent with the superoxide cluster next to R35 and R38. The accumulation of superoxide near heme 2 is strongly reduced when removing the positive charges (5A mutant, Figures 3A,B and S6). The R35A and R38A variants, as well as the R30A/R108A/K134A variant, lead to a similar, although less pronounced decrease of the proximal superoxide concentration in the MD simulations as for the 5A mutant (Figures 3B and S6). In the R30A/R108A/K134A variant, the removal of these positively charged residues in the MD simulations leaves the heme propionic groups unshielded, which leads to an inward flip of R35 and R38 toward the heme propionates, and loss of superoxide binding (Figure 3D,E). Moreover, the in silico R108A and K134A variants (Figure 3C) have a minimal effect on the superoxide distribution, while the R35A and R38A variants, result in a loss of the main peak in the superoxide distribution profiles (Figure 3C), which still feature an overall high superoxide concentration near the P-side binding site.

To test the results of our molecular simulations, we introduced mutations in the central cationic residues and spectroscopically assessed the electron transfer activity of both the solubilized and liposome- reconstituted SOO (see Methods). We followed the overall heme reduction levels of the enzyme variants, in the presence of enzymatic superoxide production by xanthine oxidase and hypoxanthine, as described previously.^18,21^ Consistent with the predictions from our simulations, mutation of all five arginine residues to glutamines (5Q) or introduction of the triple mutation (3Q – R30Q/R108Q/K134Q) lead to a drastic decrease of the overall enzyme reduction by >50% (Figure 3F). Introduction of two negatively charged amino acids into the binding pocket, e.g., the R30D/R108D (2D) variant, with an intact R35/R38 superoxide binding site, also results in a strongly lowered heme reduction, while the substitution of arginine with lysine residues (3K – R30K/R108 K/K134R) had only a minor influence on the heme reduction level, suggesting that the overall charge of the amino acids is key for the superoxide binding. The overall effects were similar for solubilized and liposome-embedded enzymes, but somewhat more apparent in the latter for the K to Q mutations, and more so in the 5Q than in the 3Q variant. Taken together, this indicates that the positive amino acids are necessary to attract superoxide to the membrane, consistent with our MD simulations (Figure 2A).

To explore the effect of the mutations in the superoxide binding site on the relative superoxide:quinone oxidoreductase activity of SOO (forward reaction), we measured the competition of xanthine oxidase produced superoxide between SOO and a superoxide indicator in the presence of WST-1, a tetrazolium salt that reacts with superoxide to formazan, while the quinone pool was regenerated by purified *bo*_3_ oxidase (see Figure S7A,B for details on the assay). In these assays, we observe an overall similar behavior as in the steady-state reduction experiments (Figure 3F). In this regard, the relative oxidoreductase activity was severely diminished (<45%) in the quintuple glutamine mutant and upon substitution of two key arginines with aspartates, while the triple mutant was less affected (60%), and unchanged activity was observed upon substitution of the arginines with lysines (Figure 3G). Taken together, our findings show that the cationic cluster near heme 2 functions as superoxide collecting antenna in SOO.

### Mechanism of Quinone Binding and PCET Leading to Quinol Formation

A putative Q binding site, populated with a glycerol molecule in the X-ray structure, is located on the cytoplasmic (N-side) of the enzyme, at a *ca*. 6 Å edge-to-edge distance from heme 1. This site comprises multiple histidine residues that could act as proton donors for the quinone reduction (Figure 1C). Under Michaelis–Menten conditions of the forward reaction, a *K*_m_ of ∼400 nM was found for ubiquinone Q_1._^21^ To study the molecular principles of quinone binding and reduction, we modeled a ubiquinone-4 molecule in different redox states (see Methods), and probed the dynamics within the binding site (see Table S1 for a list of simulations of modeled redox/protonation states).

**Figure 4:**
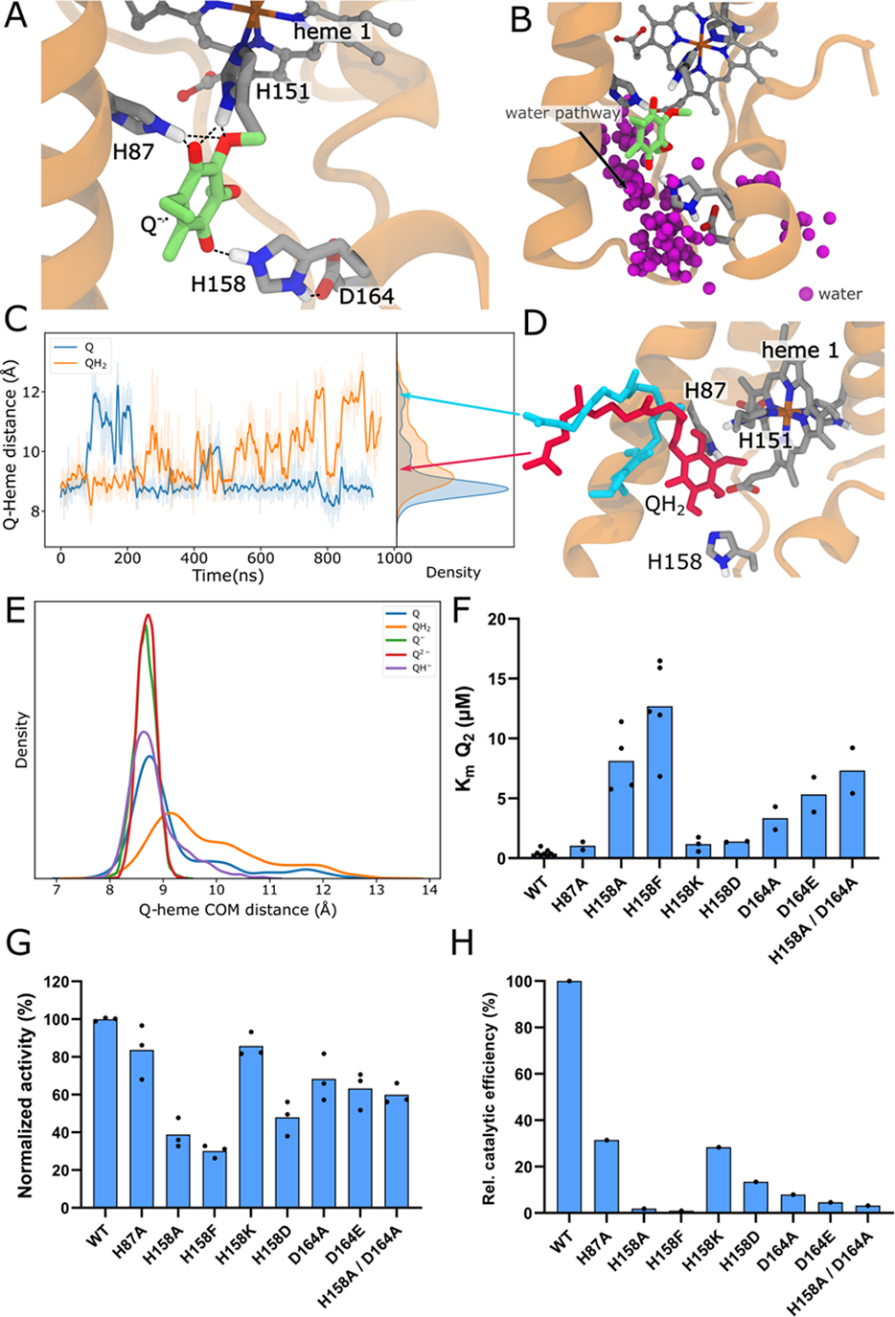
Exploration of the quinone binding site. (A) Overview of the quinone binding site in MD simulations (simulation S3). The Q forms hydrogen bonds with H151 and H158, which in turn forms an ion-pair with D164. The figure shows the semiquinone state, with hydrogen bonds established also with H87, and a strong interaction with H151 and H158. See Supplementary Figure S5 for distance distribution during simulation in different redox and protonation states. (B) Hydration of the Q site during the MD simulation (overlay of waters from 16 evenly spaced snapshots from 800 ns MD (simulation S3). A water wire connects the Q site from the cytoplasm bulk water, and could provide protons for reprotonation upon quinol formation or directly for the Q reduction. (C) Center-of-mass (COM) distance between the Q headgroup and heme 1 during simulations with oxidized Q and reduced QH_2_ (simulations S1, S6). While the Q shows some transient unbinding events, the QH_2_ shows multiple binding and unbinding events. (D) Snapshot of QH_2_ (simulation S6) in “bound” (in red) and “unbound” (in blue) conformations, with respective distances shown by the arrows in panel (C). (E) COM distances for Q-heme 1 in different redox states. The Q^·–^ and Q^2–^ states, corresponding to the first and second electron transfer to quinone without proton transfer remain strongly bound to the Q site during the MD simulations. (F, G) Binding constant *K*_m_ of Q_2_ for WT and Q site variants (F), and the steady state activity of the forward reaction by measuring the decrease in formazan rate formation upon addition of 100 nM of different variants of SOO as in ref (21). The rates were normalized relative to the WT. Mutations of H87, H158, and D164 perturb the activity, with H158 mutations having the largest impact. None of the variants fully abolish the activity suggesting that SOO can employ other pathways, such as the putative water wire shown in panel B. The binding constants show the importance of both H158 and D164 for the Q binding. See Supplementary Figure S7 for a schematic overview of the assay. (H) Catalytic efficiencies (*k*_cat_/*K*_m_) of mutants relative to the WT.

During the MD simulations (Table S1), the quinone headgroup forms hydrogen bonds with the nearby histidine residues, H151, a ligand of heme 1, and H158, which in turn forms an ion-pair with D164 (Figures 4A and S8). The oxidized Q remains overall rather stable during the microsecond MD simulations in the binding pocket, while a quinol (QH_2_) molecule, partially dissociates from the binding pocket (Figures 4C,D and S9). These findings are supported by previous biochemical experiments of SOO,^21^ which showed that QH_2_ has a 25-fold higher Michaelis constant (*K*_m_) relative to the oxidized Q. Interestingly, we find that the binding of Q affects the stability of the H158-D164 ion pair. In this regard, states with a Q bound show a stable ion pair, while both the *apo* as well as the QH_2_ bound state, in which H158 is deprotonated, lead to a weakening of the hydrogen bond and opening of the ion pair (Figure S8B). In contrast, the anionic semiquinone (Q^·/–^) species remains highly stable in the binding pocket (Figure 4A), particularly when H87 is doubly protonated (HisH^+^), and leading to a tight hydrogen bond with Q^·/–^ (Figure S8C) together with a stabilization of the H158-D164 ion-pair (Figure S8B). Our electrostatic calculations (Figure S10) suggest that while the p*K*_a_s of H158 and H87 in the crystal structure of the *apo* state are 8.6 and <4, respectively, the p*K*_a_ of H87 strongly depends on its sampled conformation in the MD simulations. In this regard, H87 prefers a neutral state (Nε protonated form) when the residue is in contact with R63 (Figure S10C), while the protonated form (HisH^+^) is preferred upon hydrogen bonding to the Q. Reduction of Q to semiquinone further stabilizes the cationic states (HisH^+^) of both histidine residues (Figure S10).

Our mutagenesis experiments show that the Q binding affinity and oxidoreductase activity (Figures S7A and 4F–H) are indeed strongly affected by the H158-D164 ion pair. In this regard, mutation of H158 with nonpolar residues (H158A, H158F) resulted in a large increase of the *K*_m_ for Q_2_ (WT: 0.4 μM; variants 8–13 μM). Similarly, mutation of D164 to alanine and glutamic acid also resulted in a significant increase of the *K*_m_ (5 and 3 μM), the latter indicating that the length of the side chain affects the ion-pair with H158 (Figure 4F). Interestingly, for the H158A/D164A variant, the observed effects on *K*_m_ in the single mutants were not cumulative, indicating that the removal of both residues is less harmful. The residual activity of these mutants could arise from the extension of the water-mediated proton pathway from the cytoplasm (Figure 4B). The relative catalytic activities of WT and quinone site mutations at saturated quinone concentrations were determined using the WST-1 competition assay (Figure S7A,B). We find that the H158A and H158F variants resulted in a reduction of the oxidoreductase activity (39% and 30% of WT activity, respectively, Figure 4G). This large decrease could result from steric effects, while other mutations (H158D, H87A, D164A, D164E) resulted in ca. 50–80% of the WT activity. Surprisingly, the H158A/D164A double variant showed ∼60% activity, but the mutations significantly lower the *K*_m_ of the reaction, supporting the importance of the ion-pair for the catalysis.

Reduction of Q to QH_2_ requires two electrons, which are stepwise transferred, from superoxide via heme 2 and heme 1, together with two protons. Based on the stable interactions of Q during our MD simulations, H158 and H87 could function as potential proton donors in the PCET reactions. However, we also observe the formation of a water wire from the N-side bulk to the Q-site (Figure 4B), which could act as an alternative proton uptake pathway or provide a route for the reprotonation of the active site after QH_2_ formation (Figure 4B), and partially rescue the activity in the H158A/D164A double variant (see above).

To probe the mechanistic principles of the PCET reactions linked to the QH_2_ formation, we next performed QM/MM molecular dynamics (QM/MM-MD) simulations and free energy calculations to explore the bond-formation and bond-breaking reactions using a density functional theory (DFT)- based QM/MM treatment of the PCET reactions. To this end, we first studied the proton transfer dynamics of Q in the oxidized (Q), semiquinone (Q^·–^), and double reduced (Q^2–^) states, without modeling the coupled electron transfer from heme 1 (Table S2, QM/MM models 1–3). When the quinone is in the fully oxidized Q state or in the semiquinone (Q^·–^) state, we observe no proton transfer reactions from the nearby histidine residues during the simulation time scale. In stark contrast, the Q^2–^ species results in an instantaneous proton transfer from both H158 and H87 (Movie 2), supporting that these residues could serve as proton donors in the quinol formation and that the second electron transfer from heme 1 to Q, is coupled to transfer of both protons. Next, to probe the electron–proton coupling during electron transfer from heme 1, we expanded our QM/MM model (Table S2, QM/MM model 4) and determined the two-dimensional free energy landscape of this PCET reaction by a finite temperature QM/MM string method (Figures S11 and S12). To this end, we optimized three possible reaction pathways: In pathway 1, we studied a concerted proton transfer in which both histidines simultaneously (synchronously) transfer their protons to Q; while in pathways 2 and 3 we explored a consecutive, i.e., stepwise (asynchronous) mechanism involving proton transfer from H87 followed by H158, or vice versa.

**Figure 5:**
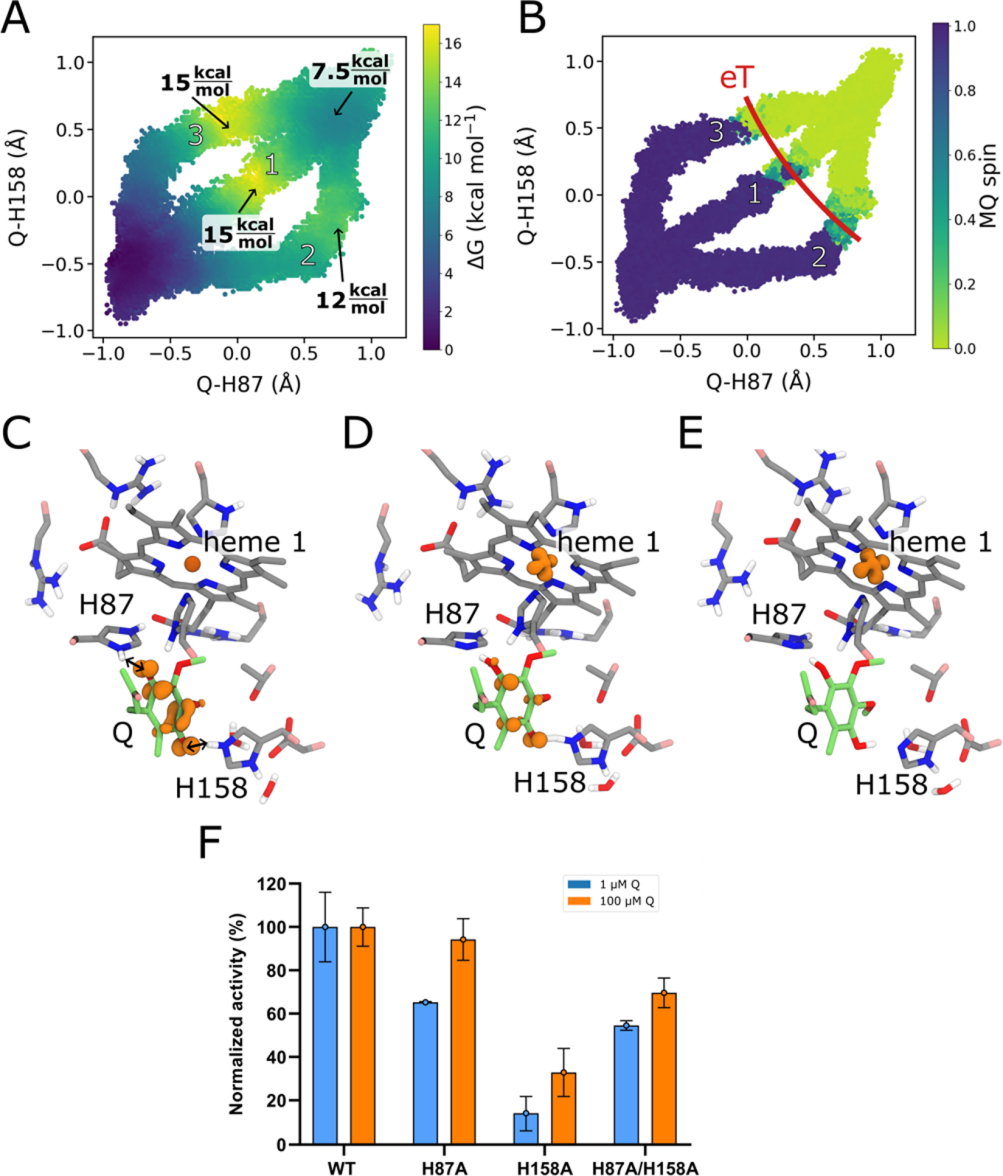
Exploring the PCET mechanism for quinone reduction. (A) 2D**-**free energy profiles from QM/MM finite temperature string simulations for the quinone reduction along three putative pathways 1–3 (numbers in white). The stepwise pathway 2 with an initial proton transfer from H87 followed by proton transfer from H158 and electron transfer from heme 1 shows the lowest free energy barrier of ca. 12 kcal mol^–1^, while the reverse order (pathway 3, H158 followed by H87) and the concerted pathway 1 are 3–4 kcal mol^–1^ higher in energy. (B) Analysis of the spin populations along the three reaction pathways, shows that the second proton transfer couples to electron transfer from heme 1 to form QH_2_. The approximate location of the electron transfer processes is marked with a red line. (C–E) Reactant, transition state and product structures representing the favored reaction pathway. Spin densities, shown as orange volumes, support the electron transfer from heme 1 to the Q. Only the QM region is shown for visual clarity. (F) Relative oxidoreductase activity of the H87A, H158A and H87A/H158A relative to WT SOO at a fixed concentration of 1 μM (blue) and 100 μM (orange) Q.

Among the explored reaction mechanisms, we obtain the lowest free energy barrier of 12 kcal mol^–1^ (comparable to a rate of 40 μs, see Methods) along a stepwise pathway (Pathway 2), where a proton is transferred from H87 to Q^·–^ followed by a concerted (simultaneous/synchronous) proton transfer from H158 with an electron transfer from heme 1 (Figure 5A,B and Movie 3). This local PCET reaction step is overall endergonic (Δ*G* ∼ 7.5 kcal mol^–1^), but it could be thermodynamically driven by the electron transfer from O_2_^·–^ (*E*_m_ = −160 mV) to Q (*E*_m_ = +90 mV) (Δ*G* = −5.7 kcal mol^–1^), and entropic (*T*Δ*S*) contribution coupled to the quinol release to the membrane pool (but see the Discussion section). The free energy barriers along the other stepwise pathway (Pathway 3, proton transfer from H158 followed by H87) as well as the concerted pathway (Pathway 1) are 3 kcal mol^–1^ higher that would result in a slower rate of ∼5 ms (based on transition state theory). In the former reaction (Pathway 3), the second proton transfer from H87 to Q^·–^ also couples to an electron transfer from heme 1 to Q^·–^ (Figure 5A,B) as in the kinetically preferred reaction mechanism (Pathway 2). However, in the concerted mechanism, both protons are transferred simultaneously to Q^·–^, together with the electron transfer from heme 1 (Figure 5A,B). Despite the stepwise/concerted pathways explored, we identify only a single barrier along the QM/MM free energy profiles (Figure 5B), suggesting that the obtained reaction pathways can be described as asynchronous/synchronous.

To validate our free energy profiles, we studied the entire two-dimensional free energy surface along the H158-Q and H87-Q reaction coordinates (Figure S13). These findings further support the stepwise (asynchronous) pathway identified with the QM/MM string simulation method with H87 acting as the initial proton donor followed by a PCET from H158 and heme 1 to Q^·–^, despite the intrinsic DFT (Figure S14) and statistical sampling (Figure S12) errors could affect the relative energetics.

To experimentally probe the effects of the identified residues, we assessed the superoxide:quinone oxidoreductase activity of the WT, H87A, H158A, and H87A/H158A double variant at nonsaturating and saturating quinone concentrations of 1 and 100 μM Q_1_, respectively. These experiments further support that while H158 and H87 indeed have important functional roles, the single point variants still have a residual activity (Figure 5F) that could be achieved by proton uptake, e.g.*,* by the water wire connected to the N-side bulk, consistent with results from our MD simulations (Figure 4B). In this regard, we find that replacement of H158 has a more drastic effect than replacing H87 also at saturating quinone concentrations. Moreover, in the double mutants, the addition of H87A acts partially as a second site suppressor, bringing the activity level to that of the H87A variant (Figure 5F).

## Discussion

Our multiscale simulations in combination with functional assays and mutagenesis experiments show that SOO harvests superoxide molecules from the bulk solvent by a patch of positively charged residues close to heme 2, consistent with previous suggestions.^18,21^ We show here that R35 and R38 act as superoxide collecting antennas that concentrate superoxide to a binding site, ca. 17 Å away from heme 2, making direct electron transfer from this position to the heme rather slow (800 s^–1^ based on electron transfer theory, see Methods). Instead, this transient docking site channels the superoxide further to the nearby cluster next to R30, R108, and K134 at a distance of around 9 Å from heme 2 (Figure 2C), enabling fast electron transfer (*k* ∼ 10^7^ s^–1^).

Our combined findings suggest that R30, R108, and K134 are important for shielding the negatively charged propionic groups of the heme that allow R35 and R38 to attract superoxide from the bulk solvent. Interestingly, similar positively charged patches are also found in other superoxide scavengers.^14,30,31^

Our MD simulations show that the quinone substrate binds in a cavity on the cytoplasmic side, in a position similar to that of a glycerol molecule observed in the crystal structure.^17^ In the MD simulations, the Q species forms hydrogen bonds with the nearby H151, H87, and H158, which in turn establishes an ion-pair with D164, while reduction of Q by PCET from H87 and H158 and heme 1 leads to opening of the D164-H158 contact that results in the quinol release from the binding pocket. In this regard, our mutagenesis experiments and activity measurements further support the functional relevance of these residues, but also suggest that the Q site possesses some functional redundancy, as no single point mutation completely inhibits the catalysis. The strongest effect is observed at position H158, with the H158A mutation, increasing the *K*_m_ for quinone ∼20-fold, while the activity is decreased 2-fold (Figure 5F). Interestingly, this strong effect can be at least partially reversed by mutation of D164A and H87A. The former effect indicates that the H158-D164 ion pair is indeed present, and H158 prefers a cationic (protonated) state due to its high predicted p*K*_a_ of 8.6. Surprisingly, also the H87A/H158A double mutant showed activity, suggesting that the proton transfer to the semiquinone can also occur via alternate routes, e.g., by the pathway from cytoplasm to the quinone site. Similar effects have also been described in other proton wires, such as in carbonic anhydrase, where replacement of proton shuttling histidine, results in proton transfer from the bulk.^32^ In this regard, the four-helical bundle structure of SOO allows access for water molecules to the inside of the protein and could thus facilitate catalytic redundancy. This can also be seen in the X-ray structure^18^ of the protein, where a glycerol molecule binds in the Q site.

Our QM/MM free energy simulations suggest that reduction of the Q takes place by a stepwise proton transfer from H158 and H87, which couples to an electron transfer from heme 1. The one-electron reduced semiquinone species remains tightly bound to this site, which is expected to prevent the premature release of the intermediate before completion of the reaction. In contrast, the quinol species has a lower affinity as compared to the oxidized Q substrate, consistent with our measured *K*_m_ values, which could favor unbinding and exchange of the quinol product with a new substrate after the catalysis.

Our QM/MM simulations suggest that the second electron transfer from heme 1 to quinone is coupled to two proton transfer reactions. Similar observations have been made in other quinone reducing enzymes, e.g. in the respiratory Complex I,^33^ where the quinol reduction couples to proton transfer from a nearby histidine and tyrosine residue,^34^ while the PCET reactions underlying the QH_2_ oxidation of Complex III have also recently been addressed by the QM/MM free energy methods.^35^ Our functional assays show that the quinone-driven heme oxidation has a *k*_cat_ of 350 s^–1^ (Figure S7C), comparable to a catalytic barrier of around 14 kcal mol^–1^ (based on transition state theory, cf. also ref (18)), which compares well with the barriers of 12–15 kcal mol^–1^ determined by QM/MM (see also Figures S12 and S14).

Although the modeled heme-driven proton-coupled electron transfer reaction is computationally highly challenging to describe, our benchmarking calculations with different density functionals provide similar predictions of reaction barriers, suggesting that the overall results are robust. We note that the size of the modeled QM region, as well as the amount of exact exchange-correlation used in the density functionals, can affect predicted barriers in complex PCET reactions as those studied here (Figure S14). We have therefore modeled a large extended QM system with 230 atoms, embedded in a classical description of the protein–membrane-water surroundings, and based on the extensive sampling, we obtain a statistically converged 2D-free energy profile for the PCET reaction, at least locally. The convergence of the string simulations into unique pathways, suggests that there are multiple minimum reaction pathways. Extensive sampling, currently limited by the large QM/MM models, employed DFT methodology, and definition of reaction coordinates/initial path (see Methods), is generally expected to converge into a single string, as found in other application.^68^ Potential energy scans along the final string pathway suggest that entropic effects contribute by *ca*. 3 kcal mol^–1^ to the free energy barriers (Figure S14). However, taken together, we expect that the intrinsic errors of the methodology (Figures S12 and S14), introduce comparable effects for all explored pathways, suggesting that the asynchronous pathway is kinetically preferred.

**Figure 6:**
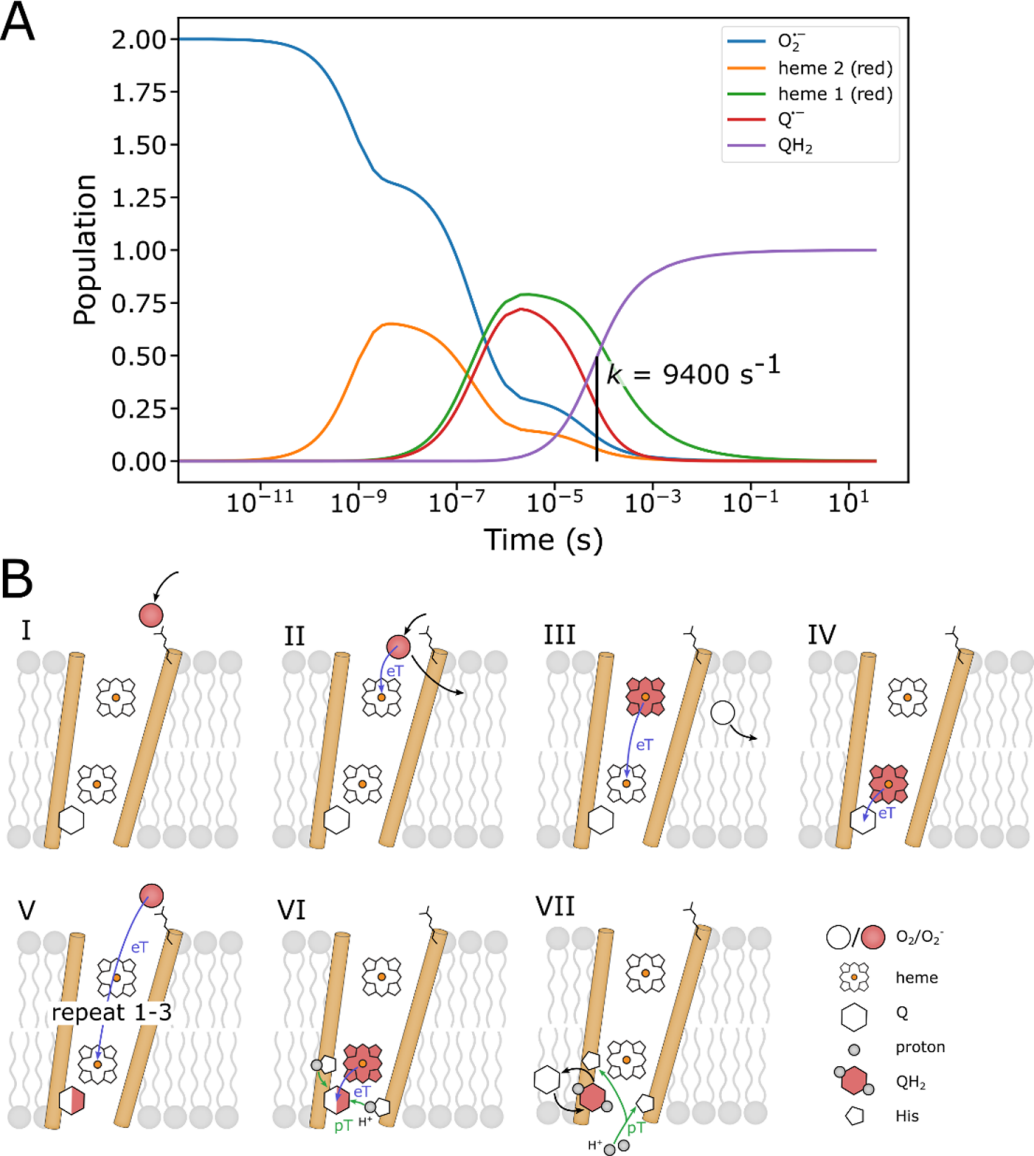
Putative charge transfer mechanism of SOO. (A) Kinetic model of the charge transfer reactions in SOO. Charge transfer rates were estimated by combination of experimental and calculated values of redox potentials and elementary rates (see Methods). The model predicts that QH_2_ is formed on μs time scales, while the O_2_^·–^ oxidation and semiquinone forms on the 10^7^–10^8^ s^–1^ time scales. (B) Schematic overview of the proposed mechanism of SOO. Oxidized cofactors are shown as empty shapes, while reduced cofactors are shown in red. The oxidized quinone molecule–blank coloring, Q^·–^ – half-filled, Q^2–^ – filled. (1) Superoxide binds close to R35/38; (2) the superoxide diffuses close to the heme 1, enabling fast electron transfer, while the resulting oxygen moves into the membrane, preventing back reactions. (3) Electron transfer from heme 2 to heme 1; and (4) from heme 1 to quinone forms a semiquinone intermediate; (5) steps 1–3 are repeated to rereduce heme 1; (6) second electron transfer from heme 1 couples to proton transfer from H168 and H87 to form QH_2_. (7) QH_2_ formation triggers opening of the H168-D164 ion pair that lowers the binding affinity and favors exchanges of an oxidized Q from the membrane pool. The water wire extending into the Q site reprotonates the histidine residues for the next reaction cycle.

To gain kinetic insight into the complete charge transfer reactions of SOO, we estimated electron and proton transfer rates based on combination of experimental redox potentials and simulation data into a kinetic master-equation model (Figure 6A, see Methods). The model predicts a rapid (ps–ns) electron transfer from superoxide to heme 2 followed by a ca. 100 ns interheme electron transfer process. Under the studied conditions, the first Q reduction step is fast (100 ns), while the re-reduction of heme 1 drives this equilibrium toward the reduced semiquinone species. Based on our QM/MM simulations, the rate-limiting step of the process is the final PCET reaction that forms QH_2_, which is roughly 2 orders of magnitude slower than the other processes and leads to a predicted overall rate of QH_2_ formation that is estimated at ca. 9400 s^–1^ in the studied conditions. Experimentally, we mimicked this situation by partially reducing SOO with one electron per enzyme, which according to the measured redox potentials^21^ is expected to populate mostly heme 1. We mixed the enzyme with either of the two substrates Q_1_ and O_2_ and followed the heme oxidation kinetics using stopped-flow experiments (Figure S7B,C). Both substrates rapidly oxidize the enzyme, but Q_1_ is ∼6-fold faster than O_2_ under these conditions, showing a kinetic advantage of the forward reaction over the backward reaction that would produce superoxide. In this regard, we note that the initial charge transfer from superoxide to quinone takes place on a much faster 10^7^ s^–1^ time scale, and could support a significantly faster, diffusion-limited rate of SOO under certain conditions (cf. also ref (18)). Such reactions could be enhanced by the local partitioning of the Q species and O_2_/superoxide into the bulk/membrane regions. Although the coupled assays relying on coreconstitution of SOO with xanthine oxidase and quinol oxidase were used here to study the superoxide-driven Q reduction, it remains experimentally challenging to control the exact amount of generated superoxide used for the activity assays. In this regard, pulsed radiolysis experiments could provide future possibilities to quantify the thermodynamics and kinetics of the reaction under highly controlled conditions.

Based on our combined findings, we propose the following mechanism for the SOO (Figure 6B): (I) In the first step, superoxide is captured by R35 and R38 and moves to a binding site ca. 17 Å from heme 2, and (II) from where the superoxide diffuses to a closer binding position that enables electron transfer to heme 2. The resulting molecular oxygen then diffuses into the adjacent nonpolar membrane to potentially prevent the back reaction. (III) The electron is then transferred on the nanosecond time scales to heme 1, from where (IV) the electron moves to the bound Q, reducing it to anionic semiquinone species, which remains tightly bound at the active site, until (V) a new superoxide molecule provides a second electron via heme 2 to heme 1. (VI) The formation of quinol is driven by PCET from the nearby histidine residues together with electron transfer from heme 1 to the semiquinone. (VII) Dissociation of the H158-D164 ion pair reduces the affinity of the quinol for the site, followed by (VIII) diffusion of the quinol into the membrane, reprotonation of the active site residues via water wires, and binding of a new quinone species.

Although the predicted energetics suggest that the superoxide-driven quinol formation is thermodynamically feasible under standard conditions, the overall respiratory conditions in vivo raise questions. In this regard, we note that the overall superoxide concentration in *E. coli* is expected to remain below 10^–10^ M under normal conditions,^36^ while the bacteria is also highly efficient in taking up oxygen from its environment.^37^ Assuming a μM cellular oxygen concentration in membranes as in mitochondria,^38^ this leads to an upshift of the oxygen/superoxide redox potential from −160 mV to ca. + 80 mV (59 mV × log_10_([O_2_^·–^]/[O_2_])), while accounting for the local concentration increase in the superoxide at the proposed superoxide collecting antenna (a factor of 10–15), would downshift the potential of O_2_/O_2_^·–^ coupled to roughly 0 mV (vs NHE, Figure 3A). The effective driving force for O_2_^·–^ → Q is thus expected to be around −100 mV under these conditions. In this mode, it is important to also note that electrons and protons are transferred against the PMF, which would limit the forward reaction membrane potentials of ca. 100 mV. Beyond these conditions, e.g., at higher membrane potentials, SOO is thermodynamically expected to favor the backward reaction, and lead to the production of superoxide. We note, however, that the forward reaction is expected to be thermodynamically feasible in the large intestine of mammals, where oxygen is scarce and superoxide levels can be higher, due to the host immune response and elevated ROS concentrations as a result of oxidative bursts in neutrophiles or macrophages.^39^

## Conclusions

Our combined findings provide detailed mechanistic insight into the function of SOO. We described how a positively charged arginine cluster (R30, R108, K134) on the P-side of the enzyme shields the negatively charged heme propionic groups, while nearby arginine residues (R35, R38) attract and concentrate superoxide to enable efficient electron transfer. We also showed how residues in the Q site facilitate substrate binding and enable the PCET catalysis that lead to quinol formation on a rapid (μs) time scale. Our integrative multiscale QM/MM free energy simulations and atomistic molecular dynamics simulations, site-directed mutagenesis and spectroscopic assays in proteoliposomes provide key insight into the charge transfer mechanism and more generally, a blueprint to understand the function of minimal oxidoreductase modules.

## Material and Methods

### Classical Molecular Dynamics Simulations

A simulation model was built based on the X-ray structure of superoxide oxidase (PDB ID: 5OC0(18)), which was embedded in a 100 × 100 Å 1-palmitoyl-2-oleoyl-*sn*-glycero-3-phosphocholine (POPC) membrane. The system was solvated with TIP3P water molecules and neutralized with NaCl (ca. 30 Na^+^/ 40 Cl^–^), corresponding to concentration of *ca*. 150 mM. Depending on the modeled state, 10 Cl^–^ ions were replaced by 10 molecules of O_2_^·/–^, and resulting in system comprising ca. 70,000 atoms (Figure 1). Ubiquinone-4 (Q_4_) was modeled in a putative Q-binding site, at the position of an experimentally resolved glycerol molecule. To this end, the Q_4_ was modeled in a binding pose that established hydrogen bonds with His158, His151, and His87. Q_4_ was used to enhance the sampling of ligand conformations, which can be limited by the slower relaxing isoprenoid tail of Q_8_, the endogenous Q species of *E. coli*.

Initial protonation states were determined based on Poisson–Boltzmann continuum electrostatics (PBE) calculations in combination with Monte Carlo (MC) sampling of the 2*^N^* protonation states in SOO, as implemented in the adaptive Poisson–Boltzmann solver^40^ (APBS) and Karlsberg+.^41^ In this approach, the atoms were modeled as point charges with a van der Waals radii, and embedded in a continuum with a polarizable dielectric medium with an ε set to 4, while the bulk water was modeled with ε = 80. A probe radius of 1.4 Å was used for detecting solvent boundaries, together with an ionic strength of 150 mM. The PBE calculations suggested that H158 is doubly protonated (HisH^+^), consistent with its putative role as a proton donor for Q. In accordance, other amino acids were modeled in their standard protonation states, except for His87, which was also modeled in its doubly protonated state in some simulations (Table S1), to probe its potential role as another proton donor in the Q-site. The membrane mimicking the *E. coli* lipid composition (75% POPE, 20% POPG, 5% cardiolipin) was created using the CHARMM-GUI membrane builder,^42^ while the pure POPC membrane was built with VMD.^43^

Atomistic molecular dynamics simulations were performed using NAMD v.2.12–2.14 and NAMD v.3α9^44^ with the CHARMM36m^45^ force field and custom DFT-derived parameters for the quinone and heme cofactors. The simulations were conducted in an *NPT* ensemble at *T* = 310 K and *p* = 1 atm using Langevin dynamics and a Nose–Hoover Langevin piston and with a 2 fs integration time step and using the SETTLE algorithm to constrain covalent hydrogen bonds. Long-range electrostatic interactions were modeled using the particle mesh Ewald (PME) approach. Cofactors and functional residues were modeled in different redox/protonation states to model the catalytic cycle (Table S1). The system was minimized and initially equilibrated for 6 ns with restraints (5 kcal mol^–1^ Å^–2^) on the backbone and cofactors, followed by 2 ns with restraints on the C_α_ atoms, followed by free unbiased simulations. VMD v.1.9.3/1.9.4^43,46,47^ and UCSF ChimeraX 1.5^48^ were used for visualization, while VMD v1.9.3/1.9.4 and MDAnalysis 2.4.3^49,50^ were used to analyze the trajectories. The radial distribution function of superoxide was measured using atom C2D of heme 2 as the center. Edge-to-edge distance distributions exclude hydrogen atoms, except for the Q-heme distance, which shows the distance between the Q-headgroup center-of-mass and the heme-Fe. Note that due to periodic boundary conditions, superoxide is able to freely diffuse between the peri- and cytoplasmic sides of the membrane.

### QM/MM Free Energy Calculations by Multidimensional String Method

To probe the energetics of the PCET reactions in SOO, hybrid quantum/classical (QM/MM) simulations were performed on a truncated, spherical model system with a radius of ca. 35 Å, centered on the Q headgroup, created based on a selected snapshot from the classical MD simulation S7. The QM region comprised ca. 230 atoms, including heme 1, Q, H13, H87, H151, H158, R59, R63, R169, D154, T166 and two waters (Table S2, QM/MM model 4), while the MM region comprised ca. 29,000 atoms (Figure S11). The Arg residues were truncated between the C_β_ and C_γ_ atoms, while other amino acids were cut between the C_α_ and C_β_ atoms and Q was truncated between the C9 and C11 atoms. The resulting boundaries between the QM and MM regions were treated with a link atom approach. The classical region was described with the CHARMM36m force field in combination with in-house DFT parameters for the cofactors. The QM region was modeled at the B3LYP-D3^51,52^ level with the def2-SVP(H,C,N,O)/def2-TZVP(Fe) basis set^53^ and using the resolution of identity approximation.^54,55^ The six-coordinated heme was modeled in the low spin state, based on similar spin states in other heme proteins.^67^ The QM/MM system was minimized for 300 steps with a Newton–Raphson optimizer as implemented in CHARMM, followed by QM/MM molecular dynamics (QM/MM MD) performed at *T* = 310 K, with a 1 fs integration step. Nonlipid molecules within 10 Å of the QM region were allowed to move, while the rest of the atoms were frozen. All QM/MM simulations were performed with the CHARMM^56^/Turbomole7.5.1^57,58^ interface.^59^ A smaller QM region (Movie 2), comprising the Q, H87, H158 and D164 (Table S2, QM/MM models 1–3), was also employed to probe the proton transfer dynamics in various redox states. To benchmark the effect of the employed DFT level, single point energies along a potential energy pathway were recalculated using different functionals (Figure S14).

### Finite Temperature String Method

A variant of the finite temperature string method^60^ was developed and implemented to probe the free energy landscape of the PCET reactions along the proton and electron transfer coordinate. To this end, initial PCET pathways were constructed with 12/19 steps (concerted/nonconcerted pathways) along the reaction coordinates defined as,
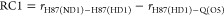

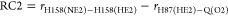
equally spaced along the pathways. The initial points were minimized for 100 steps at the QM/MM level before initiation of the production runs. Each string iteration comprised short (100–200 fs) restrained QM/MM simulations, with harmonic force constants of *k* = 50–200 kcal mol^–1^ Å^–2^ on RC1 and RC2, followed by estimation of the drift in the RCs along each string point, and fitting of a new string pathway using either a fourth order polynomial (concerted pathway), or splines (nonconcerted pathways). Both interpolation approaches gave similar results, but the spline interpolation resulted in smaller fitting errors along the nonconcerted pathways. Equally spaced points along the updated reaction pathway were generated by iteratively searching for points along the arclength between the two consecutive points, followed by simulation of a new string iteration based on the fitted points. The procedure was repeated until the pathways converged, defined as the variation in the sum of squares and maximum deviation between each pathway *i* and the previous pathway *i* – 1 varied by less than 0.008 and 0.05 Å^2^ for at least three iterations. Free energies along the converged pathways were sampled using force constants of 200 kcal mol^–1^ Å^–2^ (Figure S11), with each window sampled for 1 ps, resulting in a total sampling of 56 ps. The unbiased free energy landscape was reconstructed using the two-dimensional weighted histogram analysis method,^61^ the Multistate Bennett Acceptance Ratio,^62^ as well as the Variational Free Energy Profile method,^62^ yielding similar results. See Figure S12 for convergence of the string simulations, the free energy profiles, and overlap of the sampled RCs. QM/MM-umbrella sampling simulations were also conducted on 13 × 13 2D grid along RC1 and RC2, resulting in a total of 169 windows and 68 ps of sampling. Each window was constrained with a force constant of 200 kcal mol^–1^ Å^–2^. The unbiased 2D landscape was recalculated using the same approach as in the string method.

### Electron Transfer Rates and Kinetic Models

Electron transfer rates (*k*_*ij*_) in SOO were estimated using the Moser–Dutton ruler^63^ based on,
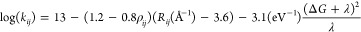
1

In this regard, mean distances and the rate of superoxide binding were estimated from MD simulations, while using standard reorganization energies (λ = 0.7 eV) and packing densities (ρ = 0.76), with *R*_*ij*_ values of 9 Å, 11 and 6 Å for the superoxide–heme 2, heme 2–heme 1 and heme 1 – Q pairs, respectively. The driving forces were based on measured redox potentials for the hemes^21^ (−8/+100 mV) and Q (+90 mV), as well as the estimated superoxide potential based on our calculations (0 mV, see Discussion/Figure 3A). For the second electron transfer to Q, the PCET energetics from QM/MM were converted into a rate constant using transition state theory, with pre-exponential factors of *k*_B_*T*/*h* = 6 ps^–1^ and neglecting barrier recrossing effects. The estimated rates were employed to generate a kinetic master equation model of the overall charge transfer by,
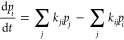
2

The master equation was integrated numerically using COPASI.^64^ The final PCET reaction from heme 1 to Q was modeled as an irreversible reaction to account for unbinding and dissociation of the QH_2_ to the membrane pool. The overall turnover of the enzyme was calculated from the half-time (*t*_1/2_) of QH_2_ formation using the relationship *k* = ln 2/*t*_1/2_.

### Electrostatic Calculations

p*K*_a_ values were determined by combining continuum electrostatics calculations with Monte Carlo sampling of protonation states. To this end, equally spaced (every 10 ns) frames were selected from the MD trajectory, and the system was truncated to include the protein, cofactors, as well as lipids within 5 Å of the protein. The surroundings were described as a continuum with a dielectric constant of 4 for the protein medium and 80 for the bulk water, with an ionic strength of 100 mM KCl. The p*K*_a_ calculations were performed using Karlsberg+^41,65^ and APBS.^40^

Q binding energetics along the MD trajectories were estimated based on a molecular mechanics/Poisson–Boltzmann solvation model (MM/PBSA) model. In this regard, equally spaced snapshots were selected every 2 ns and the system was truncated to include the entire protein as well as the cofactors and lipids within 10 Å of the protein. The Q was truncated to a Q1 and defined as the solute, while the rest of the system comprised the solvent. Both the solvent and the solute were modeled as a polarizable continuum with a dielectric constant of 10 and explicit point charges. The binding free energy was determined using a thermodynamic cycle, combing solvation free energies and coulomb interaction arising from the point charges as implemented in APBS v3.4.1.^40^

### Expression and Purification of SOO

The pcybB-His plasmid, which encodes the wild-type version of the cybB gene with a C-terminal 8xHis tag, was used as a template to insert nucleotide substitutions that resulted in single or multiple point mutations into the *E. coli* cybB gene. Either custom synthesis (Gene Universal) or site-directed mutagenesis was used to introduce these alterations. In *E. coli* BL21(DE3) pLysS cells, the expression of wild-type CybB and all mutant variants was performed as previously described.^18,21^ Briefly, cells were harvested 4 h after induction with 0.2 mM IPTG and lysed at least twice using a Maximator High Pressure Homogenizer HPL6 (Maximator AG, Switzerland) in the presence of DNase I (Merck, Germany), several antiproteases, and lysozyme. Upon ultracentrifugation, the membrane pellet was resuspended and diluted to a concentration of 10 mg mL^–1^. After solubilizing the diluted membranes in 1% OGNG (Anatrace) for an hour at 4 °C, the insoluble material was extracted using ultracentrifugation. CybB was eluted using 100 mM histidine after the resultant supernatant was added to nickel charged Profinity IMAC resin (Bio-Rad, USA). The sample was hereafter placed onto a Superdex 200 Increase 10/300 GL column. Fractions containing CybB were pooled, concentrated on a 50 kDa MWCO concentrator and snap frozen in liquid nitrogen.

### Reconstitution of SOO in Liposomes

The process of preparing liposomes and reconstituting CybB was carried out as outlined by Lundgren. Shortly, 5 mg/mL of *E. coli* polar extract (ECPE) lipids (Avanti) dissolved in chloroform was evaporated and dried overnight in a desiccator. The resulting lipid film was resuspended in a buffer containing 20 mM HEPES (pH 7.4), 20 mM KCl and 200 mM NaCl. Unilamellar liposomes were formed by seven freeze–thaw cycles followed by sonication on ice. For the reconstitution of CybB, 10 μL of purified enzyme (concentration varying between 100 and 200 μM) was mixed with 250 μL of the sonicated liposomes in the presence of 0.6% (w/v) sodium cholate and incubated for 30 min at room temperature. Afterward, the detergent was eliminated by running the mixture through a PD-10 gel filtration column (GE Healthcare), and the proteoliposomes obtained were stored at 4 °C and used for measurements on the same day.

### Activity Assays

The forward reaction (oxidation of superoxide) was monitored using a competition assay previously established and adapted, based on the water-soluble tetrazolium (WST-1) dye assay for SOD and SOO (Supplementary Figure S7A,B).^18,21,66^ To this end, formazan formation upon reduction of WST-1 by superoxide was monitored at 438 nm in assay buffer (100 mM sodium phosphate pH 8, 0.1 mM DTPA, 0.1 mM HPX, and 0.05% DDM) containing 50 μM WST-1, 20 μg mL^–1^ catalase, 1 or 100 μM ubiquinone Q_2_ and 60 nM *bo*_*3*_ oxidase). To compare the overall activity of the wt and variants at saturating Q_2_ concentrations, varying amounts of the enzyme was added to achieve a WST-1 inhibition between 20 to 50%. The difference between the slopes before and after SOO addition was divided by the amount of enzyme used to obtain a relative enzyme activity. Measurements were performed in a cuvette with a stirrer using Cary 60 UV–VIS spectrophotometer.

The Michaelis constant (*K*_m_) for the oxidized ubiquinone Q_2_ was determined (as described in ref (21)) using a constant amount of SOO (50 nM), while varying the Q_2_ concentration between 0 and 100 μM. The relative decrease of formazan formation rate upon addition of SOO^21^ was plotted in Prism (Graphpad), following the calculations of the apparent *K*_m_ value.

The reverse (quinol oxidation and superoxide formation) reaction was followed by monitoring superoxide production using WST-1 as before (see ref (21)). In this regard, varying amounts of prereduced ubiquinol QH_2_ was mixed in assay buffer (100 mM sodium phosphate pH 8, 0.1 mM DTPA, and 0.05% DDM, containing 50 μM WST-1). The reaction was initiated by addition of 50 nM SOO and formazan formation was followed at 438 nm using a Cary 60 UV–vis spectrophotometer. The Michaelis constant was calculated based on the initial formazan formation rates, plotted against quinol concentration, and fitted using Prism (Graphpad).

### Stopped Flow Kinetics

Kinetic measurements of the oxidation of the partially reduced WT SOO were resolved by SX20 stopped-flow spectrometer (Applied Photophysics) with a mixing time of 1 ms at room temperature. All measurements were performed in 100 mM phosphate, pH 8, 20 mM KCl, 200 mM NaCl, 0.05% DDM under completely anaerobic conditions. Anaerobicity was maintained by combining N_2_ and a glucose oxidase system as described in ref (21). During the measurements, the drive syringe A was filled with 2 μM of SOO, partially reduced by 8 μM of sodium dithionite, while the syringe B was filled with either 100 μM of anaerobic quinone Q_1_ or an aerobic buffer. The single mixing ratio was 1:1 with a drive volume of approximately 110 μL. Oxidation of reduced enzyme was initiated by addition of either substrate. The data obtained were fitted using a two-exponential model.

### Steady State Reduction of SOO

Overall enzyme reduction of WT and variants of SOO was monitored using a Cary 60 UV–Vis spectrophotometer (Agilent Technologies, USA) by measuring heme absorbance changes at 428 nm upon addition of either quinol or produced superoxide in aerobic conditions. The measurements were performed either in 100 mM sodium phosphate pH 8, 0.1 mM DTPA and 0.05% DDM, or in 50 mM Tris-HCl pH 9, 0.1 mM DTPA, 0.05% DDM when quinol or superoxide were used as electron donor. In this regard, 0.25 μM of either solubilized SOO or 50 μL of proteoliposomes was used and the reaction was started by adding either 0.01 U xanthine oxidase (bovine milk (Sigma, USA)) in the presence of 0.1 mM hypoxanthine as a superoxide source or 20 μM of prereduced Q_2_H_2_ with sodium borohydride. The complete reduction of SOO was achieved by addition of sodium dithionite and used hence to calculate the percentage of relative heme reduction.

## Supporting Information Available

MD simulations, simulation data and experiments, enzyme dynamics, calculated pKa values, and superoxide binding and quinone reduction; trajectories, MD and QM/MM setups, kinetic model, p*K*_a_ and binding energy calculation setup, as well as experimental data can be found in the Zenodo repository: 10.5281/zenodo.14164391 (PDF)
Superoxide binding in a putative binding site during MD simulations (MP4)
Quinone protonation dynamics in different redox states based on QM/MM simulations (MP4)
Mechanism of quinone reduction based on the finite-temperature string simulations (MP4)
