# Supplementary material for: Molecular Principles of Proton-Coupled Quinone Reduction in the Membrane-Bound Superoxide Oxidase
Source: J Am Chem Soc. 2025 Feb 12;147(8):6866–79. doi: 10.1021/jacs.4c17055 (PMC11869295; doi:10.1021/jacs.4c17055)
Supplement: Supplementary file 1 — ja4c17055_si_001.pdf [file ja4c17055_si_001.pdf]

Supplementary information

for

Molecular principles of proton-coupled quinone reduction in the membrane-bound superoxide oxidase

Daniel Riepl[1], Abbas Abou-Hamdan[2], Jonas Gellner[1,3], Olivier Biner[2], Dan Sjöstrand[1], Martin Högbom[1], Christoph von Ballmoos[2,*], Ville R. I. Kaila[1,*]

Content

Table S1. List of MD simulations.
Table S2. Overview of QM/MM systems.

Figure S1. Overview of the simulation system.
Figure S2. Effect of membrane composition.
Figure S3. Root-mean-square deviation during MD simulations.
Figure S4. Enzyme dynamics.
Figure S5. Electrostatic map of SOO.
Figure S6. Radial distribution of superoxide around SOO.
Figure S7. Activity assays and site-directed mutagenesis experiments.
Figure S8. Distances distributions within the quinone binding site.
Figure S9. Quinone binding energetics.
Figure S10. Calculated pK_a values.
Figure S11. QM/MM setup.
Figure S12. String method and convergence of the free energy simulations.
Figure S13. QM/MM-US of the 2D PCET reaction.
Figure S14. Benchmarking the DFT calculations.

Table 1: List of MD simulations. If not stated otherwise, H158 is modelled in the doubly protonated state, while all other residues are in their reference state. prot - protonated ($HisH^+$), deprot - Nε protonated histidine.

Simulation	O_2 (ox) or $O_2^{\bullet-}$ (red)	heme 2	heme 1	quinone	Length (ns)	Comment
**S1**	red	ox	ox	Q	1000, 2 x 400	-
**S2**	ox	red	ox	Q	800, 2 x 400	-
**S3**	red	ox	ox	$Q^{\bullet/-}$	800, 2 x 400	-
**S4**	red	ox	ox	Q^{2-}	750, 2 x 400	-
**S5**	red	ox	ox	$QH^{\bullet/-}$	750, 2 x 400	H158 deprot
**S6**	red	ox	ox	QH_2	1000, 2 x 400	H158 deprot
**S7**	red	ox	ox	Q	1000, 2 x 400	H87 prot
**S8**	red	ox	ox	$QH^{\bullet/-}$	2 x 400	H158 deprot, H87 prot
**S9**	red	ox	ox	Q	4 x 1000	*E. coli* membrane, H87 prot
**S10**	red	ox	ox	Q	1000, 2 x 400	R30A, R35A, R38A, R108A, K134A, H87 prot
**S11**	red	ox	ox	Q	1000, 2 x 400	R30N, R35N, R38N, R108N, K134N, H87 prot
**S12**	red	ox	ox	Q	1000, 2 x 400	R30A, H87 prot
**S13**	red	ox	ox	Q	1000, 2 x 400	R35A, H87 prot
**S14**	red	ox	ox	Q	1000, 2 x 400	R38A, H87 prot
**S15**	red	ox	ox	Q	1000, 2 x 400	R108A, H87 prot
**S16**	red	ox	ox	Q	1000, 2 x 400	K134A, H87 prot
**S17**	red	ox	ox	Q	1000, 2 x 400	R35A, R38A, H87 prot
**S18**	red	ox	ox	Q	1000, 2 x 400	R30A, R108A, K134A, H87 prot
**S19**	red	ox	ox	Q	1000, 2 x 400	R30A, R35A, K134A, H87 prot
**S20**	red	ox	ox	Q	1000, 2 x 400	R30A, R35A, R38A, H87 prot
**Total**					**36 µs**	

Table 2: Overview of QM/MM systems. Systems 1-3 were employed to determine the relevant Q redox for the PCET reaction involving heme 1. Based on this, System 4 was used to determine the two-dimensional free energy surface for the PCET reaction. See also Fig. S11 for a rendering of System 4.

System	Residues	Q redox state	heme 1 redox state	Non-standard protonation state
**1**	Q, H87, H158, D164, 2 waters	Q	-	H87, H158 protonated
**2**	Q, H87, H158, D164, 2 waters	$Q^{\bullet/-}$	-	H87, H158 protonated
**3**	Q, H87, H158, D164, 2 waters	Q^{2-}	-	H87, H158 protonated
**4**	Q, heme 1, H13, R59, R63, H87, H151, H158, D164, T166, R169	$Q^{\bullet/-}$	red	H87, H158 protonated

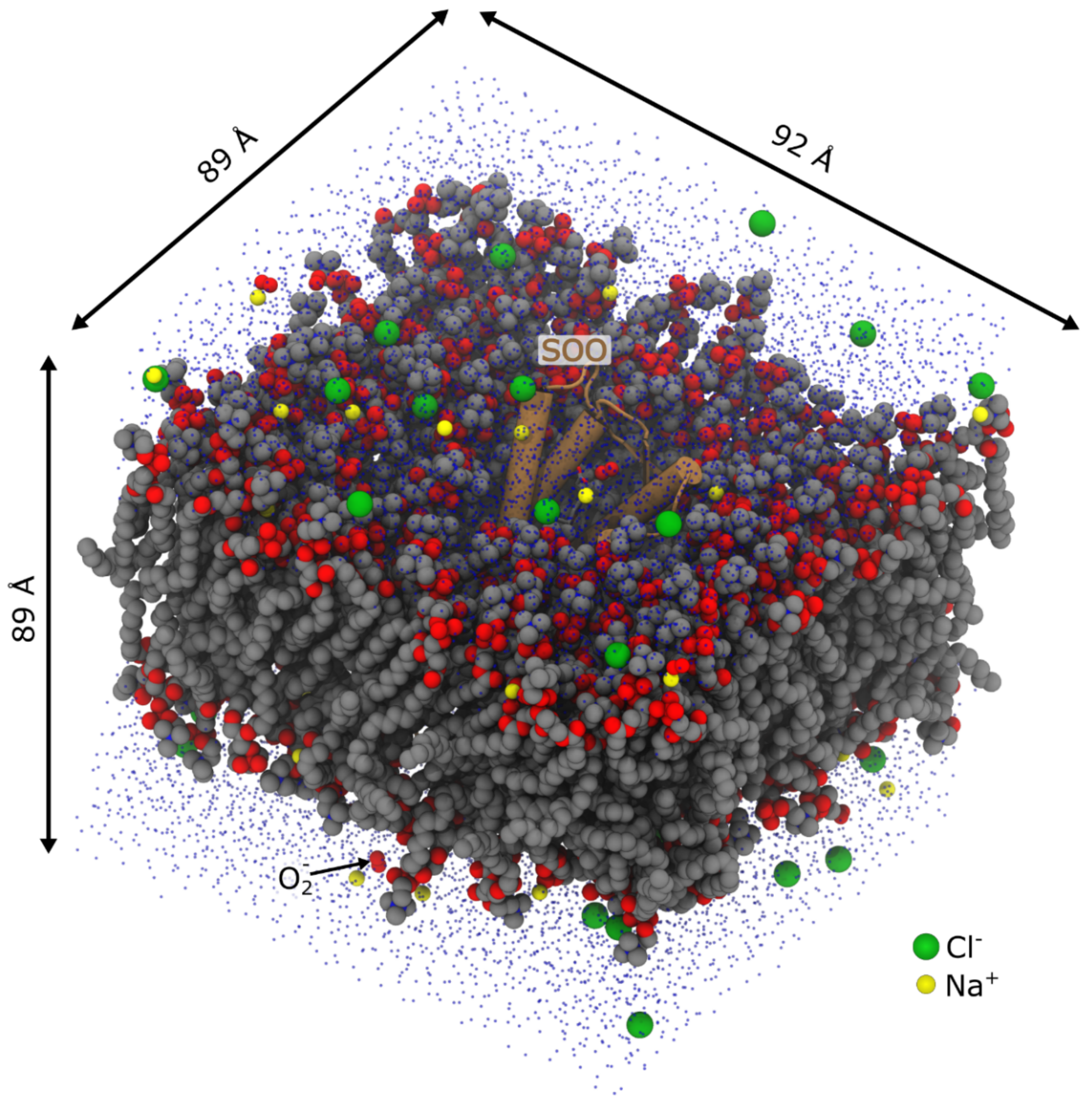

Figure S1. Overview of the simulation system. The protein is shown in cartoon representation, lipids, and ions as spheres, and water molecules as blue dots.

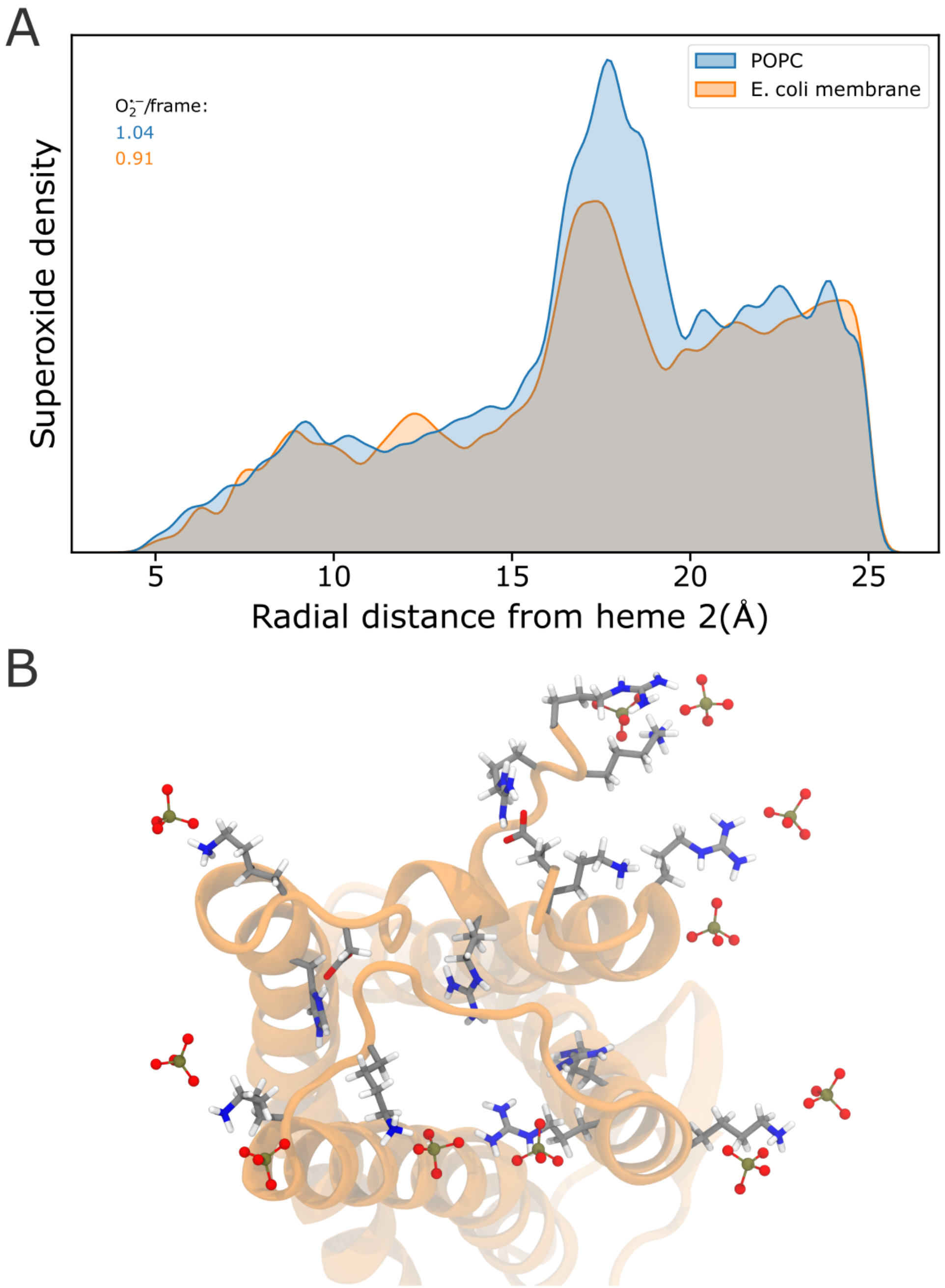

Figure S2. Effect of membrane composition. A) Radial distribution of superoxide around heme 2 in a pure POPC membrane and in a membrane that mimics the *E. coli* lipid composition (75% POPE, 20% POPG, 5% Cardiolipin). Both membranes show similar behavior, with a slight decrease in superoxide in the *E. coli* membrane, possibly due to the high amount of negatively charged lipids. **B)** Charged residues on the cytoplasmic side of the protein form transient interactions with lipid headgroups. Shown here is a snapshot from simulation S1. For visual clarity only the phosphate groups of the lipids are shown.

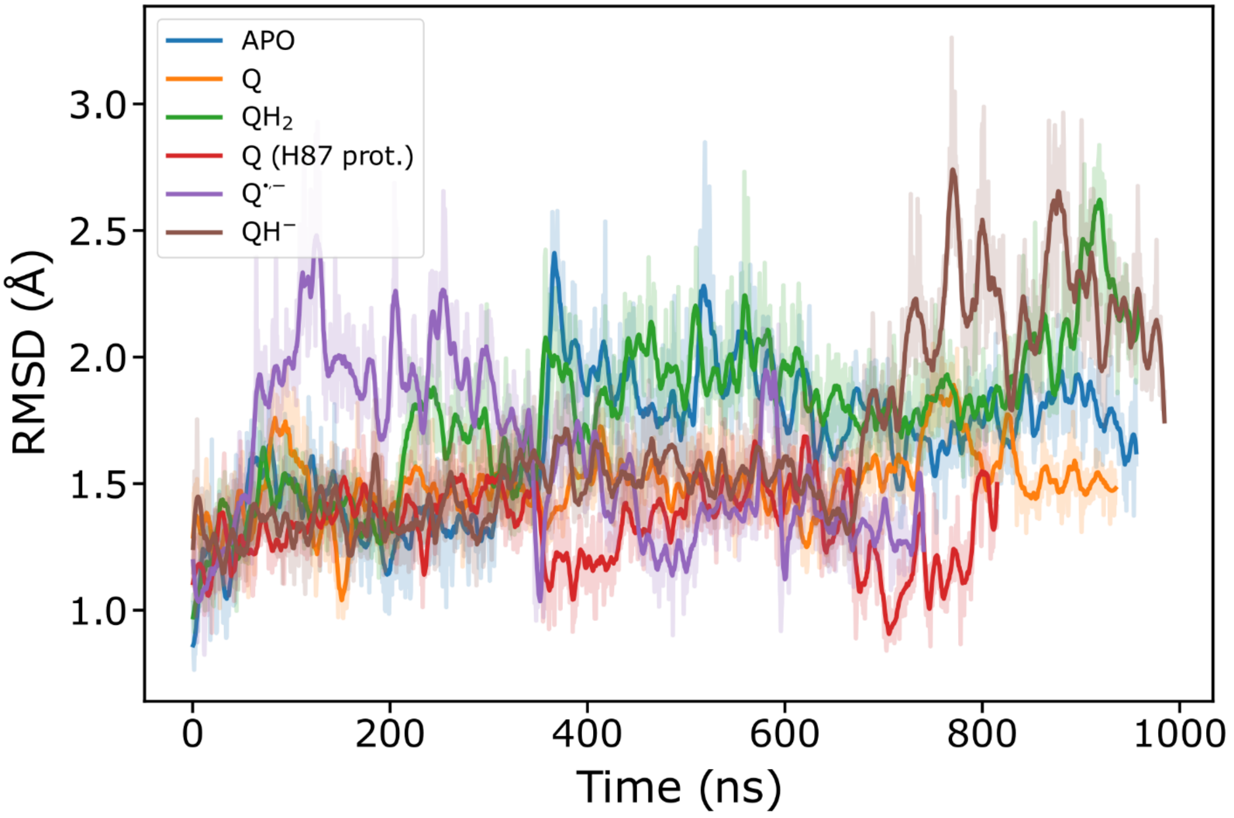

Figure S3. Root-mean-square deviation (RMSD) during MD simulations. Stability of SOO during MD simulations show a small RMSD relative to the crystal structure.

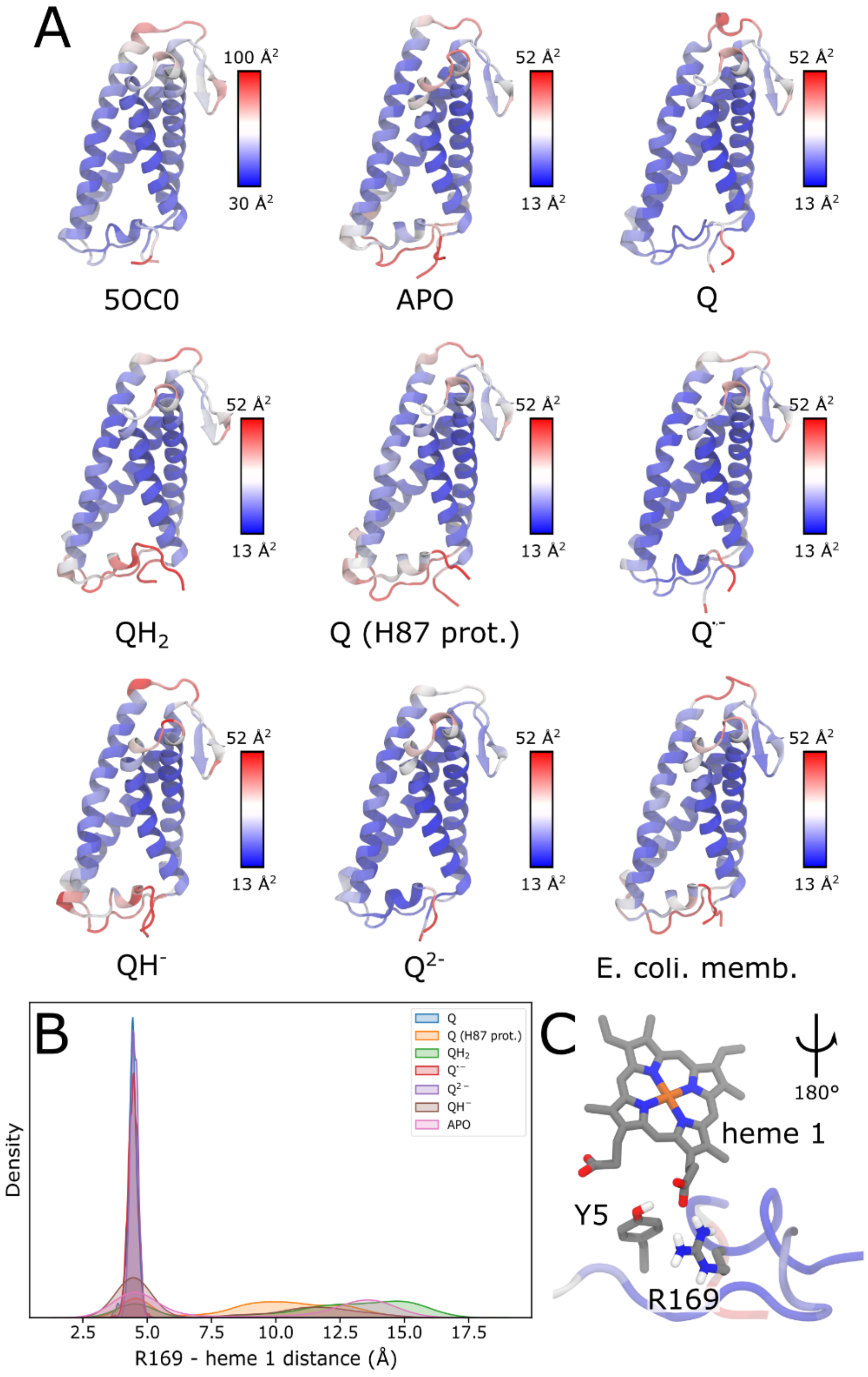

Figure S4. Enzyme dynamics. A) Comparison of calculated B-factors from MD simulations with experimental B factors from the crystal structure of SOO. Overall, the relative flexibility of different parts of the enzyme is in good agreement between the experimental structure and MD data. The core regions of the transmembrane helices are rigid. On the periplasmic side (*top*), the loop containing R35 and R38, shows a high degree mobility, while on the cytoplasmic side (*bottom*) the C-terminus is very dynamic. The flexibility of the loop and short perpendicular helix on the cytoplasmic side varies between different simulations and seems to be dependent on whether R169 forms a contact with a propionic group of heme 1 (panel B, C). **B)** Distance distribution between R169 and the heme 1 propionic group. **C)** Snapshot of the interaction between R169 and heme 1 in a simulation with Q. The nearby Y5 also interacts with the propionic group. The protein is coloured by B-factor analogous to panel A.

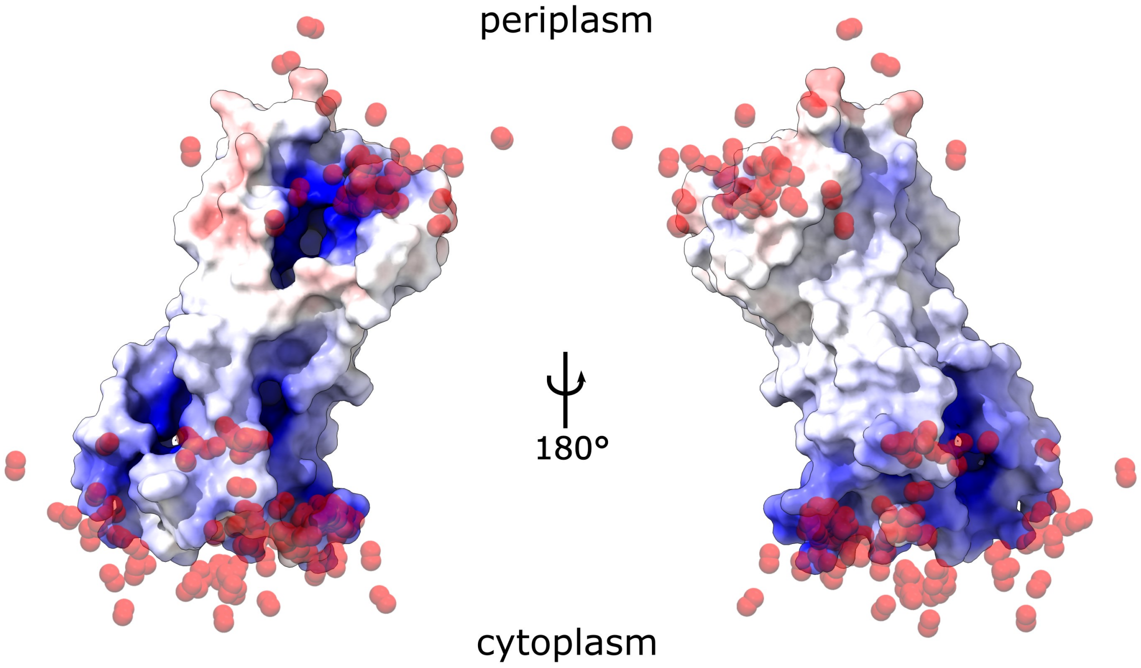

Figure S5. Electrostatic map of SOO. The periplasmic side of the protein shows a positively charged binding pocket. Superoxide positions from the MD simulations are overlayed. Note that superoxide also binds to the cytoplasmic side of the protein due to the periodic boundary conditions used in the simulations that allow superoxide to freely exchange between both sides of the membrane.

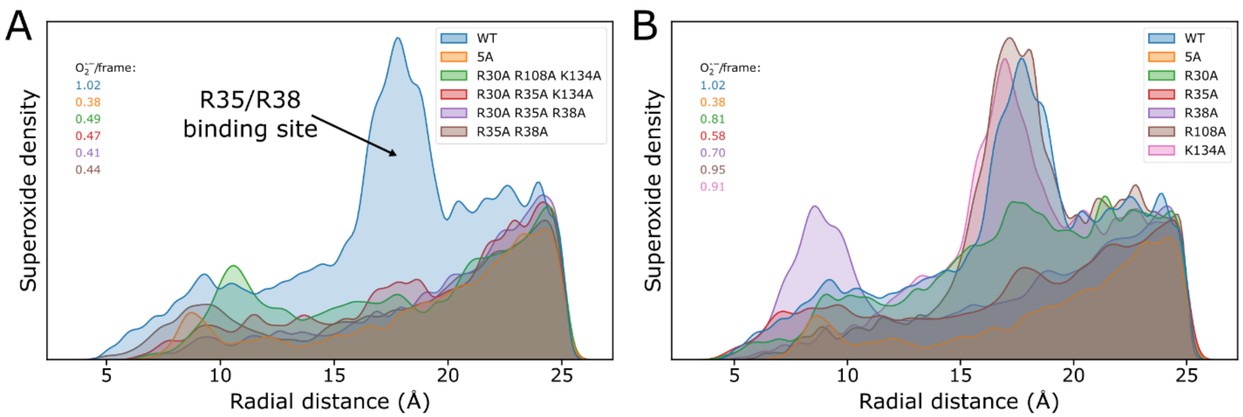

Figure S6. Radial distribution of superoxide around SOO. A, B) Radial superoxide distribution, $g(r)$, relative to heme 2 (atom C2D) in the WT and *in-silico* variants. Average number of bound superoxide per frame (integral of $g(r)$) are reported in the upper left corner. Quintuple and triple mutants show significantly lower superoxide concentrations, with loss of the main binding peak that corresponds to the R35/R38 site. Single point mutants resemble the WT closer, with the exception of R30A and R38A mutations that lead to the disappearance of the peak at *ca*. 17 Å (see also main text Fig. 3D, E).

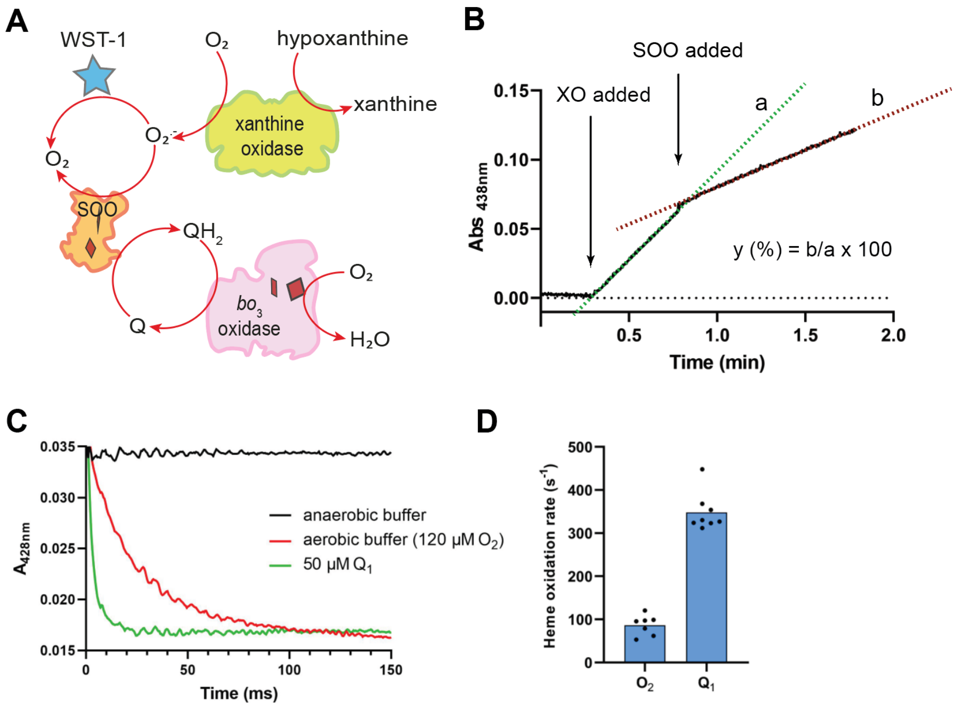

Figure S7. Site-directed mutagenesis experiments. A) Schematic overview of the coupled assay used to determine rate and binding constants for SOO. SOO and WST-1 both compete for available superoxide. The relative decrease of WST-1 formation in the presence of SOO is attributed to the SOO activity. **B)** Raw data for relative activity determination of SOO wt and mutants as described in panel **A**. Upon addition of xanthine oxidase (XO), superoxide is produced as monitored by formazan formation and observed at 438 nm. Addition of SOO leads to a competition of WST-1 and SOO and a decrease in WST-1 formation is observed (aim for reduction of 20% to 60% for reliable measurements). The relative activities can be calculated by dividing the ratio of slopes before and after addition of SOO by the used enzyme concentration. **C)** Stopped-flow kinetics following heme oxidation in detergent solubilized, partially reduced (~1 e^-) SOO if mixed with either oxidized Q_1 (50 μM) or aerobic buffer (~120 μM O_2). **D)** Results from stopped-flow kinetics as described in panel S7C from two biological replicates.

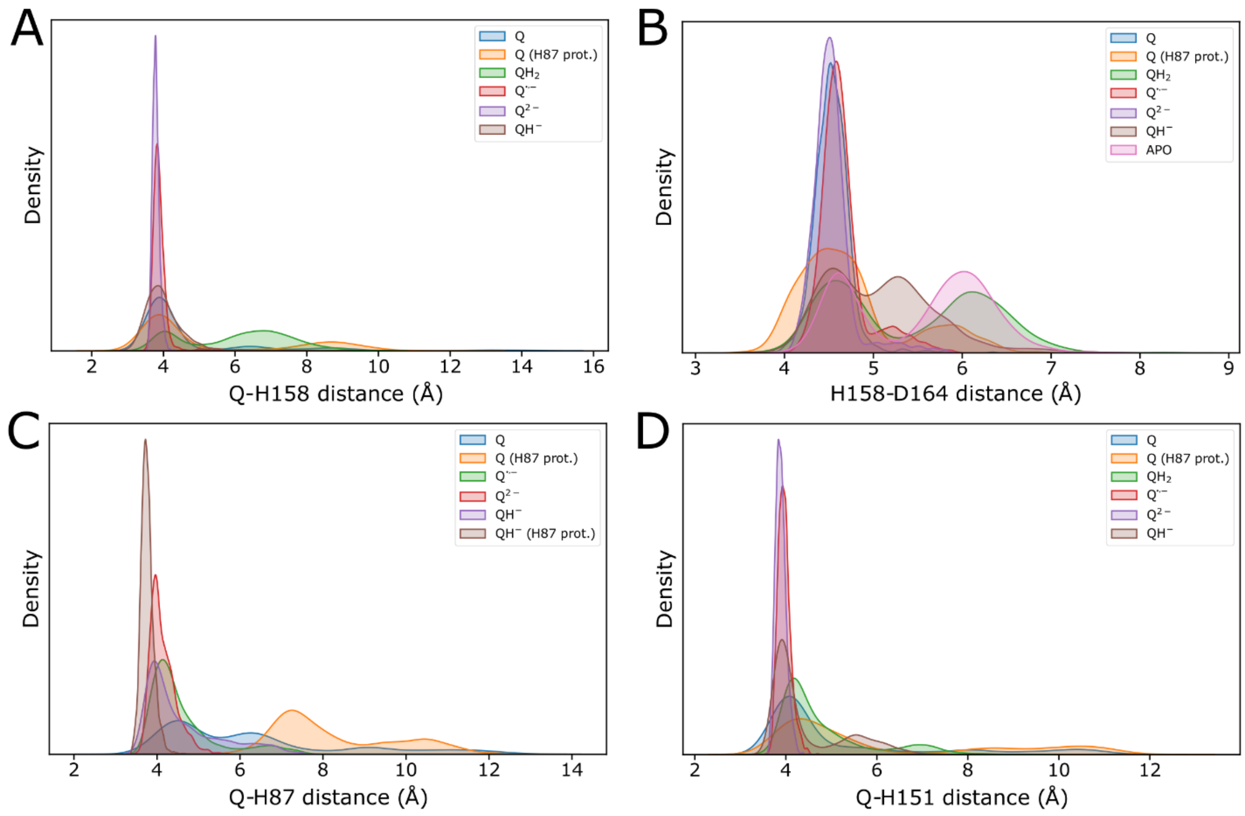

Figure S8. Distance distributions within the quinone binding site. Distances are measured using the O2/O5 atoms for Q, the center-of-mass of the histidine rings, and the CG atom of D164. **A)** Distance distribution between Q and the putative proton donor H158 in a variety of redox/protonation states. States in which a proton has been moved from H158 to Q, *i.e.*, QH^- and QH_2, show less interaction between both of them. Trajectories with H87 modelled in the doubly protonated state, also show slightly less interaction between H158 and Q. **B)** Distances between H158 and D164 show that both residues form a stable interaction with each other. This interaction is slightly disturbed when H87 is modelled protonated and strongly disturbed in the apo and QH_2 state. **C)** Distance between Q and H87 in different redox/protonation states. Interestingly, the doubly protonated H87 does not form interactions with the oxidized Q, but instead coordinates the heme propionic group. Upon modelling, a reduced Q species, *e.g.*, QH^-, H87 forms a highly stable hydrogen-bond with the Q. **D)** Distances between Q and H151. Overall, the interaction is very stable, with the exception for some unbinding in simulations with QH_2 and protonated H87.

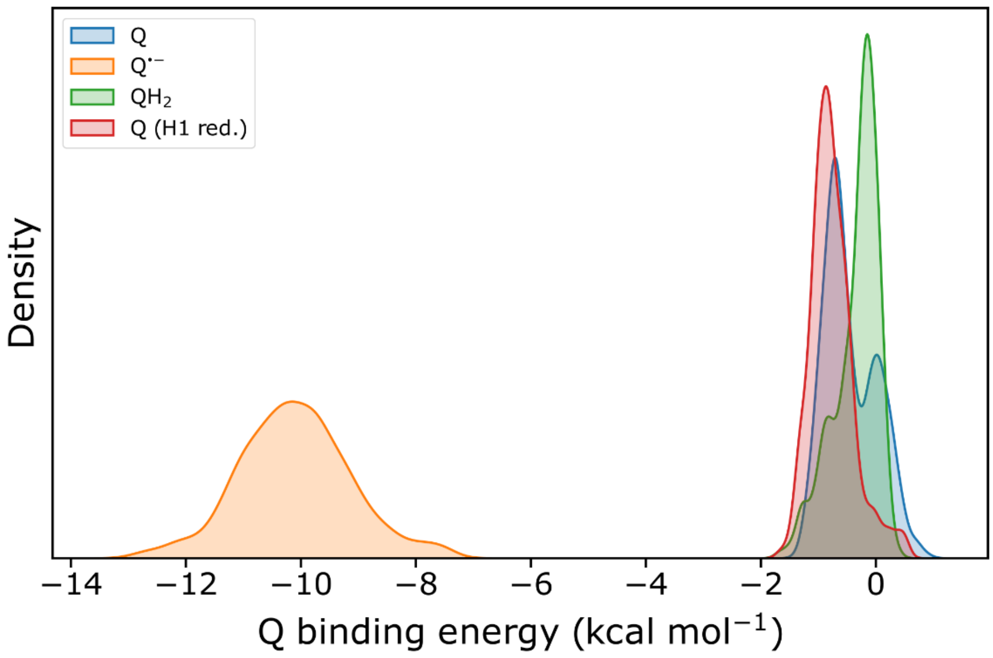

Figure S9. Quinone binding energetics. PBSA/MM binding free energies were calculated for MD trajectories with different Q redox state. The anionic semiquinone binds strongly to the active site, consistent with its stable hydrogen bonding (Figure S8), whilst the neutral Q and QH_2 have a binding affinity of a few kcal mol^{-1}. The PBSA/MM calculations further suggest that the QH_2 species binds weaker than Q (see also Figure 4E), with the reduction of heme 1 enhancing the Q affinity.

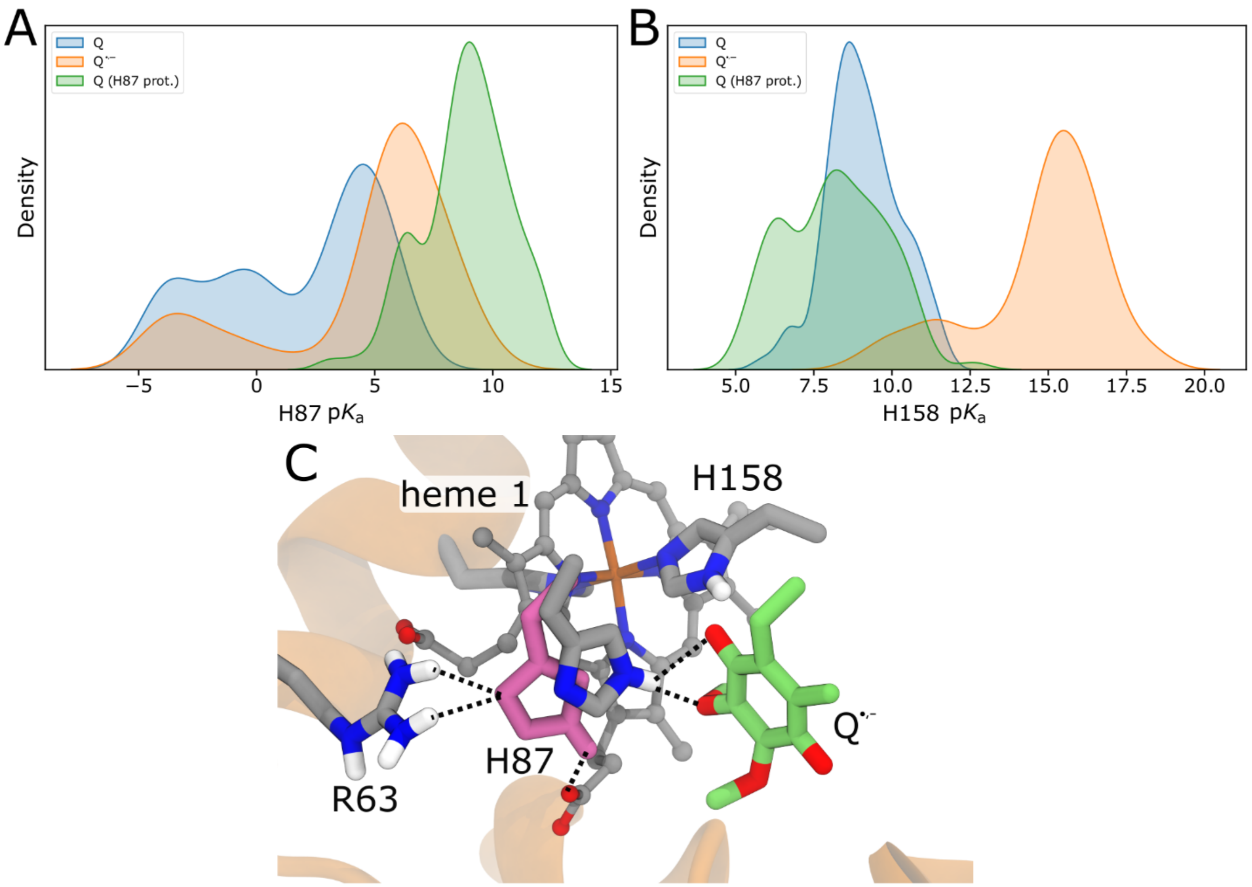

Figure S10. Calculated pK_a values. pK_a values for **A)** H87 and **B)** H158 were estimated along the MD trajectories using PBE/MC calculations. While the values significantly depend on the sampled conformation, SOO could support both the protonated and deprotonated states of H87 and H158. H158 generally favors the protonated form in pH=7, while H87 adapts different conformations to support the protonated state (green and blue curve). In both cases, reduction of Q stabilizes the protonated state of the histidines. **C)** H87 can adopt different conformations in the Q site. In one conformation (shown in pink), H87 interacts with R63, favoring the deprotonated form of H87, while in the other conformation (grey/blue), H87 interacts with the Q, which favors the protonated form of H87.

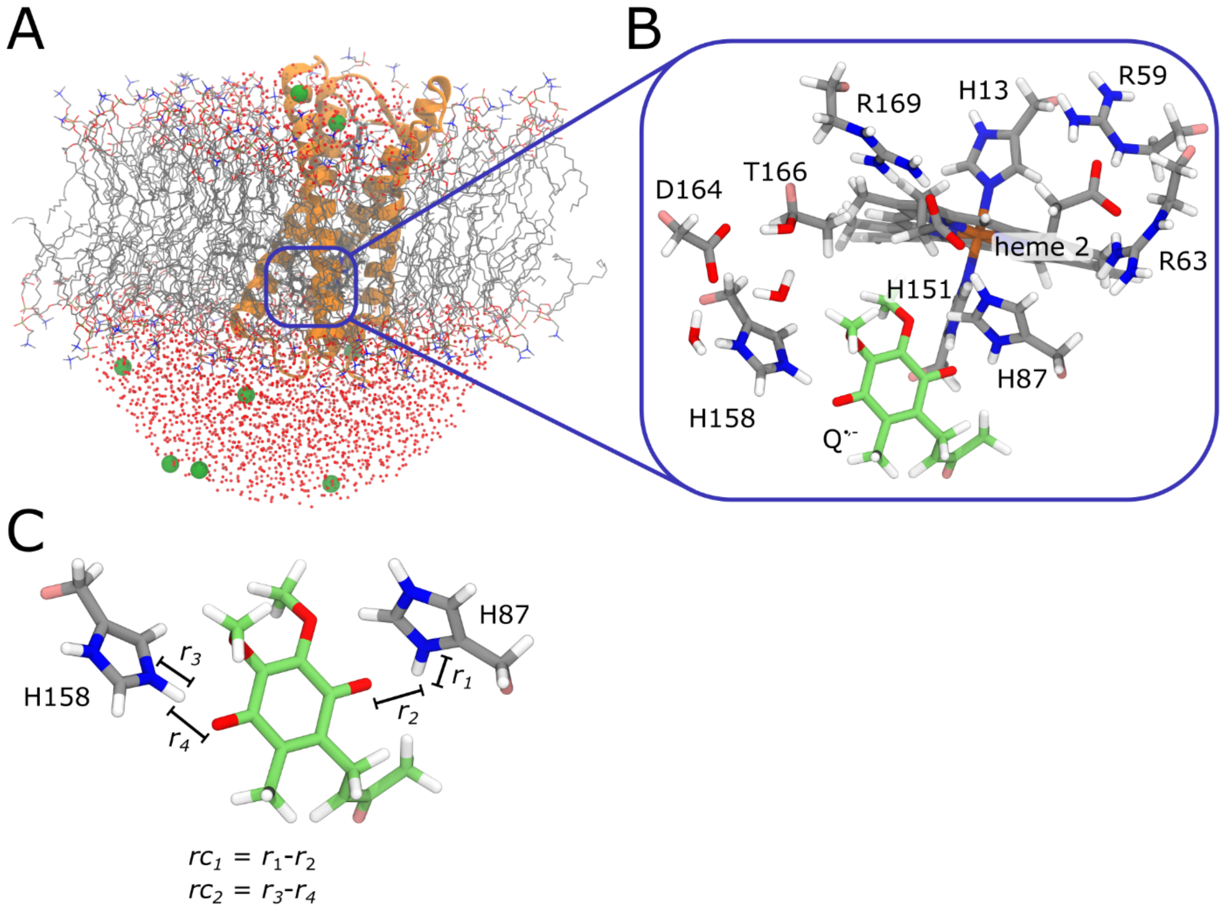

Figure S11. QM/MM setup. A) Overview of the QM/MM system for exploration of the quinone reduction process. The whole QM/MM system comprised ca. 29,000 atoms. **B)** The QM region comprised 226 atoms, with the heme in the reduced and quinone in the singly reduced state. **C)** The reaction coordinate used to model the PCET reaction, with H87 and H158 serving as the proton donors.

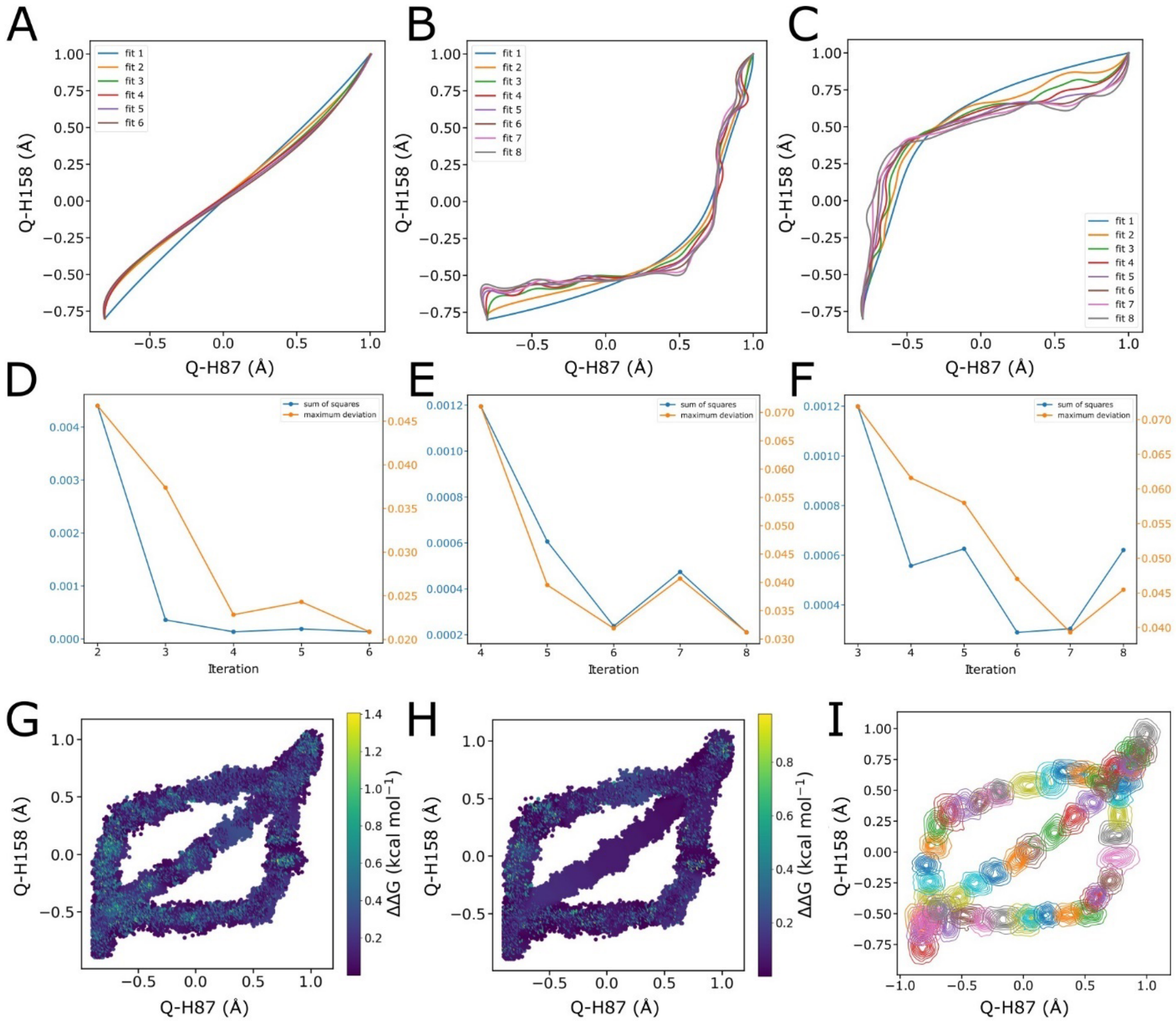

Figure S12. String method and convergence of the free energy simulations. A-C) Evolution of the string along multiple iterations. Panel **A** shows the concerted and panels **B/C** the stepwise pathways. The pathway in **A** were fitted with a 4th order polynomial, whilst the stepwise reactions were fitted with splines leading to smaller fitting errors. **D-F)** Geometrical convergence of the strings shown in panels **A-C**, respectively. The sum of squares and maximum deviation along consecutive strings are compared (see methods). **G, H)** 2D free energy difference between 600 fs and 1000 fs (panel **G**), and 800 fs and 1000 fs (panel **H**) of QM/MM sampling. The energy difference is <0.5 kcal mol^{-1} for the latter case, indicating a converged string. The concerted pathway converged in *ca*. 800 fs per window, whilst the stepwise pathway converged in ca. 1 ps/window. **I)** Overlap of sampling windows along the different pathways.

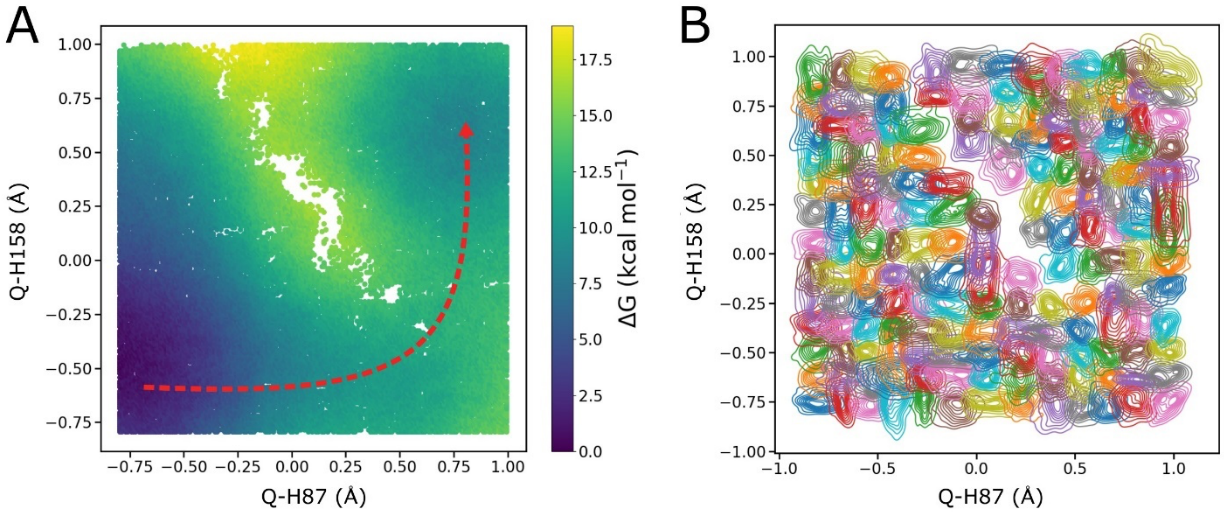

Figure S13. QM/MM-US of the 2D PCET reaction. A) 2D free energy landscape reconstructed from *ca*. 100 ps QM/MM sampling across 169 evenly spaced windows. The lowest energy pathway is marked with a dashed line, corresponding to a proton transfer from H87 followed by a concerted PCET from H158 and heme 1. **B)** Overlap of the 2D sampling, indicating high barriers along the concerted pathway (see Figure S8).

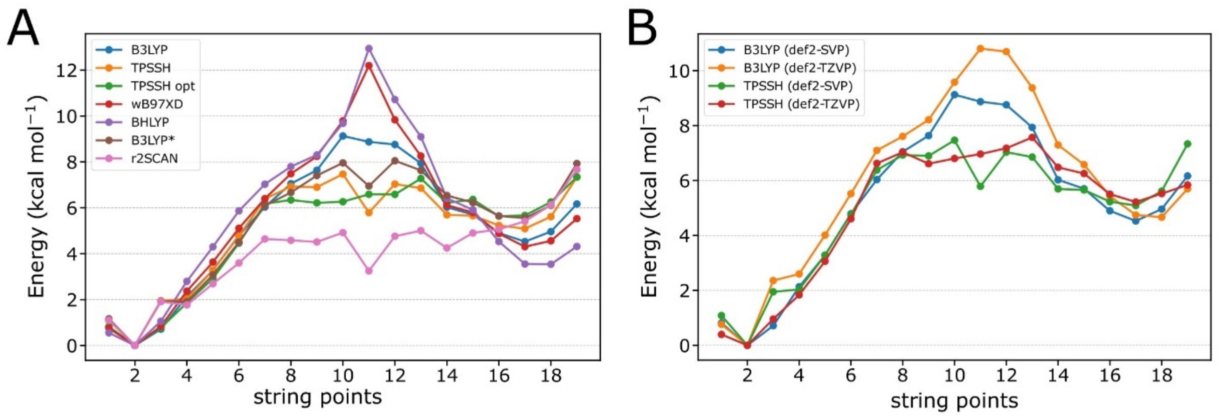

Figure S14. Benchmarking the DFT calculations. A) Comparison of the energetics of the lowest energy pathway, as identified by the string method, with different density functionals. The pathway was minimized at the B3LYP level (or at the TPSSh level for the "TPSSH opt" curve) for 200 steps followed by single point energy evaluation using different density functionals, with the DFT-D3 dispersion correction and with def2-SVP(H, C, O, N)/def2-TZVP(Fe) basis sets. The reaction barriers are sensitive to the amount of exact Hartree-Fock exchange, with a lower HF exchange leading to lower barriers as expected, while the driving force shows an opposite but smaller effect. **B)** Basis sets effects on the reaction barrier.

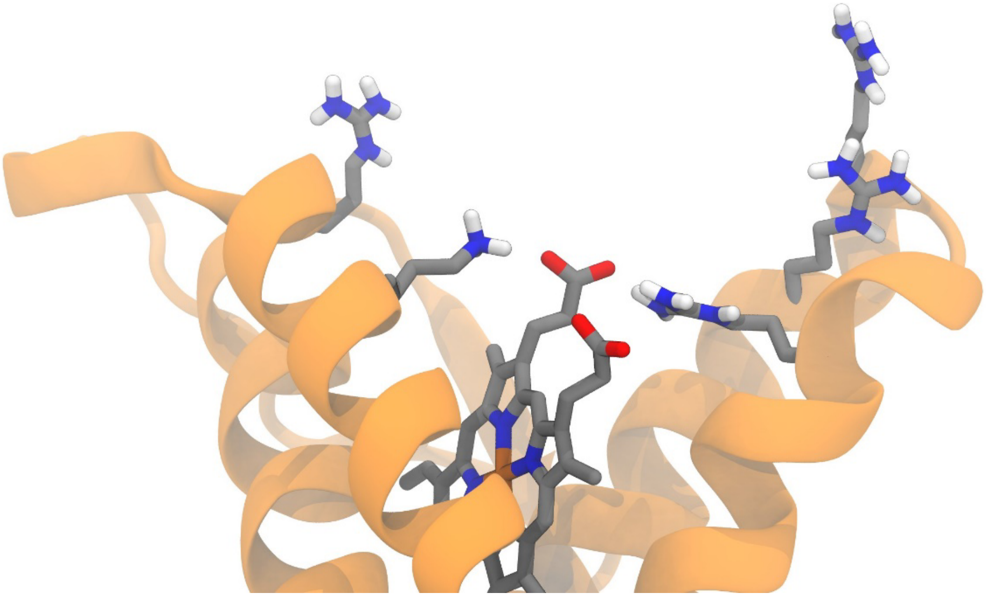

Movie 1. Superoxide binding in a putative binding site during MD simulations.

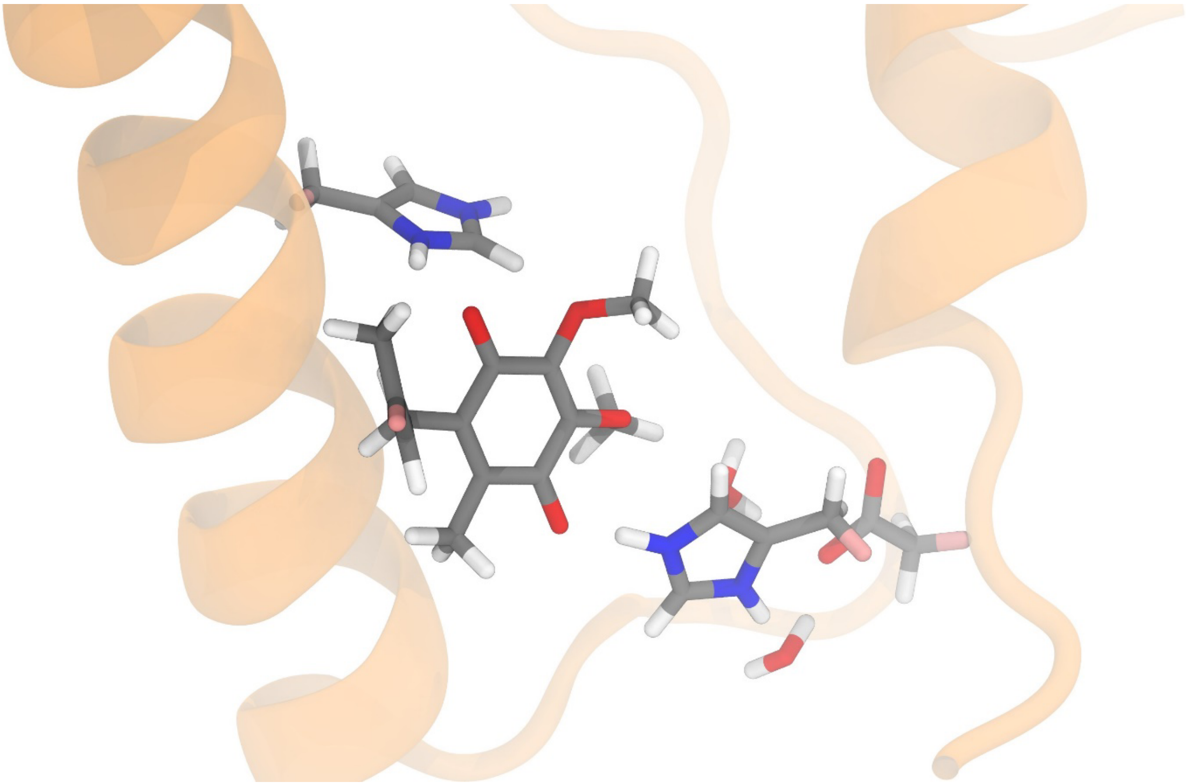

Movie 2. Quinone protonation dynamics in different redox states based on QM/MM simulations. 2-electron reduction of the Q leads to instantaneous proton transfer from the nearby histidine residues, while no spontaneous proton transfer occurs in the oxidized or singly reduced Q states.

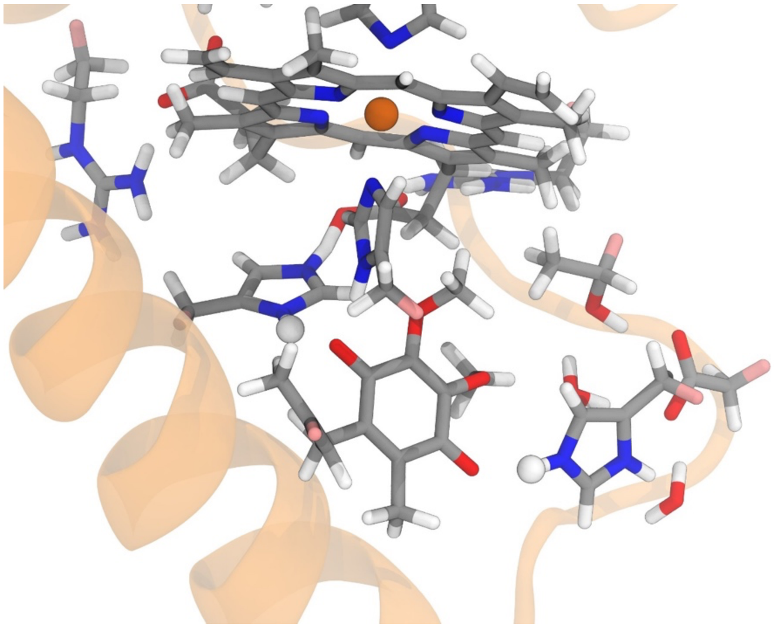

Movie 3: Mechanism of quinone reduction based on the finite-temperature string simulations.
